# Detection and genome characterisation of SARS-CoV-2 P.6 lineage in dogs and cats living with Uruguayan COVID-19 patients

**DOI:** 10.1590/0074-02760220177

**Published:** 2023-01-16

**Authors:** Yanina Panzera, Santiago Mirazo, Mariana Baz, Claudia Techera, Sofía Grecco, Florencia Cancela, Eddie Fuques, Emma Condon, Lucía Calleros, Natalia Camilo, Andrea Fregossi, Inés Vaz, Paula Pessina, Nikita Deshpande, Ruben Pérez, Alejandro Benech

**Affiliations:** 1 Universidad de la República, Facultad de Ciencias, Instituto de Biología, Departamento de Biología Animal, Sección Genética Evolutiva, Montevideo, Uruguay; 2 Universidad de la República, Facultad de Ciencias, Sección Virología, Montevideo, Uruguay; 3 Universidad de la República, Facultad de Medicina, Instituto de Higiene, Departamento de Bacteriología y Virología, Montevideo, Uruguay; 4 WHO Collaborating Centre for Reference and Research on Influenza, Peter Doherty Institute, Melbourne, Victoria, Australia; 5 Universidad de la República, Facultad de Veterinaria, Unidad de Clínica y Hospital Veterinario, Montevideo, Uruguay; 6 Universidad de la República, Facultad de Veterinaria, Laboratorio Clínico del Hospital Veterinario, Montevideo, Uruguay

**Keywords:** SARS-CoV-2, domestic animals, next-generation sequencing, serology, One Health

## Abstract

**BACKGROUND:**

Severe acute respiratory syndrome coronavirus 2 (SARS-CoV-2) infections in domestic animals have occurred from the beginning of the pandemic to the present time. Therefore, from the perspective of One Health, investigating this topic is of global scientific and public interest.

**OBJECTIVES:**

The present study aimed to determine the presence of SARS-CoV-2 in domestic animals whose owners had coronavirus disease 2019 (COVID-19).

**METHODS:**

Nasopharyngeal and faecal samples were collected in Uruguay. Using quantitative polymerase chain reaction (qPCR), we analysed the presence of the SARS-CoV-2 genome. Complete genomes were obtained using ARTIC enrichment and Illumina sequencing. Sera samples were used for virus neutralisation assays.

**FINDINGS:**

SARS-CoV-2 was detected in an asymptomatic dog and a cat. Viral genomes were identical and belonged to the P.6 Uruguayan SARS-CoV-2 lineage. Only antiserum from the infected cat contained neutralising antibodies against the ancestral SARS-CoV-2 strain and showed cross-reactivity against the Delta but not against the B.A.1 Omicron variant.

**MAIN CONCLUSIONS:**

Domestic animals and the human SARS-CoV-2 P.6 variant comparison evidence a close relationship and gene flow between them. Different SARS-CoV-2 lineages infect dogs and cats, and no specific variants are adapted to domestic animals. This first record of SARS-CoV-2 in domestic animals from Uruguay supports regular surveillance of animals close to human hosts.

Coronaviruses (subfamily *Orthocoronavirinae*, family *Coronaviridae*, order *Nidovirales*) are a recognised cause of disease in humans and animals. They are classified into four genera based on their genetic and antigenic properties: *Alpha*, *Beta*, *Gamma* and *Deltacoronavirus*. *Alpha* and *Betacoronavirus* infect mammals, while Gamma and *Deltacoronavirus* infect mainly birds.^1^

*Betacoronavirus* includes the severe acute respiratory syndrome coronavirus 2 (SARS-CoV-2), the causative agent for the coronavirus disease 2019 (COVID-19) in humans.^2^ Since March 2020, SARS-CoV-2 (subgenus *Sarbercovirus*) has been the most significant public health crisis, devastatingly affecting the global economy.

SARS-CoV-2, like other coronaviruses, has a relatively large single-stranded positive-sense RNA genome (~ 30 kb) with well-characterised open reading frames (ORFs) coding for proteins engaged in viral replication and transcription (nsp1-16) and the structure of the virion (spike, matrix, small envelope and nucleocapsid). The genome also contains ORFs for accessory proteins (3a, 6, 7a, 7b, 8 and 10) that modulate the infection process in the natural host.^3^ ^,^ ^4^

The coronavirus genome has fast evolution and high plasticity driven by point mutations, deletions and insertions (indels) and recombination. This feature contributes to the rapid transmission and global spread of SARS-CoV-2 and the emergence of new strains with new biological properties.^5^ ^,^ ^6^

SARS-CoV-2 has an ancestral zoonotic origin from bats and probably includes a hitherto unknown intermediate host.^7^ The initial outbreak in the human population occurred in December 2019 in China, and from then, the virus spread worldwide by interpersonal transmission.^8^ ^-^ ^10^ The possible anthroponotic transmission between humans and animals (production, recreation animals and domestic animals) raises awareness and concerns. It alerts health authorities to take significant measures to identify receptive species and avoid transmission cycles.^11^

As a *Betacoronavirus*, it is expected that SARS-CoV-2 transmission was restricted to mammals and that birds were refractory.^12^ Susceptibility studies indicate that several mammal species could have high levels of affinity to the virus since they present the same cellular receptor protein as humans, the angiotensin-converting enzyme 2 (ACE-2), and could be receptive to infection by SARS-CoV-2.^12^ ^-^ ^14^ The susceptibility of several mammalian species has been confirmed experimentally *in vivo* or by detecting naturally-occurring infections.^15^ ^-^ ^17^ Captive wild felids are susceptible to SARS-CoV-2, including tigers, lions, leopards and cougars.^18^ ^,^ ^19^ Minks are particularly receptive to SARS-CoV-2, evidenced by a rapid animal-to-animal transmission associated with a high mortality rate observed in several fur farms in the Netherlands and the United States.^20^ ^-^ ^22^

Cases of natural infections in domestic dogs were first reported in China in an asymptomatic Pomeranian and a German shepherd living with their COVID-posi­tive owners.^23^ Additional asymptomatic and symptomatic cases of respiratory illness were detected in dogs from owners with COVID-19.^14^ ^,^ ^24^ Positive cases in domestic cats were first reported in Belgium and later in China, the United States, Japan and European countries.^25^ ^-^ ^27^

In South America, natural infection cases occurred in dogs, cats and some wild species (https://www.woah.org/en/what-we-offer/emergency-and-resilience/covid-19/). Argentina identified four dogs and two cats, one cat with symptoms associated with COVID-19.^28^ Brazil and Ecuador also notified dog and cat cases.^29^ ^,^ ^30^ Chile and Peru reported positive symptomatic cats.^31^ ^,^ ^32^ Colombia reported the same situation in a symptomatic infected dog.^33^

SARS-CoV-2 neutralising antibodies were detected in cats in Wuhan, demonstrating infection and production of antibody response under natural conditions.^34^ All cat sera collected before the outbreak were negative, suggesting that SARS-CoV-2 infected the cat population after infecting humans. SARS-CoV-2 can be transmitted to other susceptible cats under experimental conditions in the same facility.^35^ Studies of prevalence using SARS-CoV-2 neutralising antibodies showed variable values in cats, very low in Europe and Brazil (0.002-0.5%), intermediate values in Asia (10%) and high values reach up to 31% in Peru.^34^ ^,^ ^36^ ^-^ ^38^

Based on these reports, it is now accepted that companion animals are susceptible to SARS-CoV-2, with cats being highly sensitive and might transmit the illness to other naive cats. At the same time, dogs are less vulnerable.^35^ ^,^ ^39^ Epidemiologic analysis suggests that humans were the source of SARS-CoV-2 infection in most animals.^11^ ^,^ ^40^ Human origin is particularly evident in domestic animals. The high identity of full-length nucleotide sequences of SARS-CoV-2 between dogs and cats, their owners, and the neighbouring human population indicates human-to-animal transmission.^26^ ^,^ ^41^ Genomic and epidemiological data from captive animals also support a close evolutionary relationship with the keepers’ viral strains, but the transmission route is not always established. For example, differences in the viral sequences between wild felids and nearby humans from the Bronx Zoo revealed that transmission events between susceptible species are not always straightforward to interpret.^18^

SARS-CoV-2 might also be transmitted back to people from other animals in some cases, as observed in minks that infected two farm workers and stray cats in the Netherlands.^21^ ^,^ ^42^ Recently, a possible event of cat-to-human transmission was reported in Thailand.^43^

SARS-CoV-2 infection cases in companion animals usually showed a low frequency^29^ ^,^ ^44^ and these were isolated cases, yet they are not so sporadic anymore. If there is enough research, the cases occur more often than not. 

From the perspective of One Health, the investigation of transmission networks among humans and domestic animals is a topic of global scientific and public interest. 

In this study, we detected SARS-CoV-2 in a dog and a cat host and obtained both complete genomes. Neutralising antibodies were tested against ancestral Delta and Omicron BA.1 strains.

## SUBJECTS AND METHODS

*Ethics statements -* The Animal Use Ethics Committee of the Faculty of Veterinary Medicine (CEUA-FVET) approved the experimental protocol with the number 1105/2020. Veterinarians from the Department of Small Animal Medicine conducted the clinical examination and sampling of the animals. Each owner gave their written consent and completed a data sheet with the animal data (Table). 

**TABLE:** Enumeration of dogs and cats present in the homes visited - Group 1 and group 2 samples

Household	Dogs (breed, age and sex)	Cats (breed, age, and sex)	Animals symptoms
Group 1			
3 people, 1 positive	Crossbreed, 2 years, F Crossbreed, 7 years, F	0	Healthy
2 people, 2 positives	Kelpie, 2 years, F	0	Healthy
2 people, 2 positives	Beagle, 13 years, M Teckel, 2 years, M	0	Healthy
4 people, 3 positives	Crossbreed, 15 years, F	0	Healthy
5 people, 1 positive	Crossbreed, 8 years, F Crossbreed, 5 years, F Crossbreed, 5 years, M Crossbreed, 4 years, M	Crossbreed, 15 years, F	Healthy
1 person, 1 positive	Rhodesian, 7 years, F	0	Healthy
1 person,1 positive	0	Crossbreed, 5 years, F	Healthy
1 person, 1 positive	Poodle, 10 years, F Poodle, 7 years, M	0	Healthy
2 people, 1 positive	0	Siamese, 8 months, M	
2 people, 2 positive	0	Crossbreed, 11 months, M	Healthy
1 person, 1 positive	0	Crossbreed, 1 year, M^*a*^ Crossbreed, 1 year, F	Healthy
2 people, 2 positive	Crossbreed, 15 years, F	Crossbreed, 8 years, M	Healthy
3 people, 3 positives	0	Crossbreed, 6 years, F Crossbreed, 5 years, F	Healthy
1 person, 1 positive	Crossbreed, 1 year, F	Crossbreed, 4 years, F	Healthy
2 people, 2 positive	Crossbreed, 13 years, F Crossbreed, 9 years, M	0	Healthy
3 people, 2 positive	Rottweiler, 3 years, M^*a*^ Cimarron, 3 years, F	0	Healthy
Group 2			
4 people, 2 positive	Cimarron, 5 years, F Cimarron, 3 years, F Teckel, 3 months, M	0	Healthy
2 people, 1 positive	Retriever, 3 years, M	Crossbreed, 1 year, M	Healthy
3 people, 2 positives	Crossbreed, 18 years, F	Crossbreed, 15 years, M Crossbreed, 6 years, M	Healthy
3 people, 1 positive	0	Crossbreed, 1 year, M	Epiphora, sneezing, cough
2 people, 2 positive	0	Crossbreed, 13 years, F	Healthy

The clinical signs and symptoms recorded in people with coronavirus disease 2019 (COVID-19) were anosmia (52.3%), dry cough and laryngitis (33.3%), loss of taste (33.3%), fatigue (23.8%), myalgia (23.8%), headache (23.8%) and runny nose (14.3%). Only one person required hospitalisation and 14.3% were asymptomatic. No human or animal deaths were observed during the study. *a*: severe acute respiratory syndrome coronavirus 2 (SARS-CoV-2) positive animals.

*Setting and inclusion criteria -* Thirty-nine domestic animals (24 dogs and 15 cats) from Montevideo were sampled from August to December 2020. Pet samples were collected from 20 households where at least one person was diagnosed as positive. The households were classified into two groups: group 1 samples collected within 15 days from diagnosed owners and group 2 samples collected beyond 15 days of the diagnosis. The records include the total number of animals and humans inhabiting the same home, clinical symptoms, age, sex and breed of the domestic animals (Table). 

*Clinical examination -* The clinical examination included the evaluation of the general state of health, degree of hydration, an inspection of the skin and mucosa (oral, conjunctive, genital and anal), palpation of lymph nodes (mandibular, parotid, axillary and popliteal), cardiopulmonary auscultation and rectal temperature measurement. 

*Sample collections -* Each animal’s nasopharyngeal/oropharyngeal and rectal swabs were placed in a 15 mL sterile tube containing 3 mL of viral transport medium (VTM). A blood sample (3 mL in cats and 5 mL in dogs) was also obtained; half of the blood sample was placed in a dry tube and the other half in a tube with anticoagulant [fluorinated ethylenediamine tetraacetic acid (EDTA)]. Tubes with the swabs and blood were transferred to the laboratory within 2 hours after being obtained. The VTM was aliquoted and stored at -70ºC until further analysis. Haematocrit and blood count were immediately obtained from the EDTA tubes, while blood from the dry tubes was centrifuged at 1,500 rpm for 15 minutes to get the serum, and the supernatant was aliquoted and stored at -70°C. 

To obtain the samples from the cats, animals were sedated by an intramuscular injection of 10% ketamine hydrochloride (10 mg/kg) and 1% acepromazine maleate (0.1 mg/kg), while in the dogs, this procedure was not necessary.

*Haematological analysis -* The blood count was performed on an automated haematology counter (Mythic 18 Vet Hematology Analyzer, Orphée, Geneva, Switzerland). The differential leukocyte count and cell morphology were performed immediately after the blood arrived at the laboratory through a smear and staining with the May Grunwald-Giemsa technique under an optical microscope.

Serum biochemistry was performed on a semiautomatic analyser, CB 350i (Wiener lab Group, Rosario, Argentina), determining the renal profile (urea and creatinine), liver profile (total protein, albumin, cholesterol, serum alkaline phosphatase, alanine aminotransferase and aspartate aminotransferase) and ionogram (sodium and potassium).

*SARS-CoV-2 detection -* The virus RNA was extracted from nasopharyngeal and rectal swabs using the Quick-RNA™ MiniPrep Kit Viral Kit (Zymo Research, Irvine, USA). Extracted RNA (12 µL) was reverse transcripted using random primers with Superscript II^®^ reverse transcriptase (Thermo Fisher Scientific, Waltham, USA). Real-time quantitative polymerase chain reactions (qPCR) were performed using the Specific 2019-nCoV-RdRp probe.^44^ The qPCR was carried out in a 20 µL reaction volume with 1× Hot Rox Master Mix (Bioron, Römerberg, Germany), 300 nM each primer, 1.6 µM probe, and using 1 and 3 µL of cDNA. All qPCR assays were duplicated with an internal control that detects dog and cat 18S genes.^45^ The number of PCR cycles (cycle threshold or Ct) necessary to determine positive tests is than 35. Amplification reactions were performed on the ABI Prism 7500 (Applied Biosystems, Foster City, USA). 

*Microneutralisation assay -* Microneutralisation assays, the gold standard for evaluating SARS-CoV-2 neutralising antibodies, have been performed as previously described^46^ ^,^ ^47^ with some modifications**.** SARS-CoV-2 isolates [SARS-CoV-2/Australia/Vic/01/20 (ancestral; Wuhan-1 like SARS-CoV-2)], SARS-CoV-2/Australia/Vic/18440/2021 (Delta) and SARS-CoV-2/Australia/NSW/RPAH-1933/2021 (Omicron B.A.1) were kindly provided by Dr Julian Druce from Victorian Infectious Diseases Reference Laboratory. They were passaged in Vero and Vero-TMPRSS2 cells with a culture medium containing minimal essential medium (Gibco, Thermo Fisher Scientific) supplemented with 2 mM GlutaMAX (Gibco, Thermo Fisher Scientific), 1% (v/v) penicillin-streptomycin (Gibco, Thermo Fisher Scientific), 15 mM HEPES (Gibco, Thermo Fisher Scientific) and 5% (v/v) foetal bovine serum. Vero-TMPRSS2 cells also required 200 mg Geneticin (Gibco, Thermo Fisher Scientific). All cell maintenance and cell-based assays were carried out in humidified incubators at 37°C with 5% CO_2_. Sera from the SARS-CoV-2 positive dog and cat were heat-inactivated (30 minutes at 56ºC), and serial two-fold dilutions were prepared from a 1:20 dilution. Equal volumes of serum and virus [100 TCID_50_(50% tissue culture infectious dose) of each SARS-CoV-2 variant] were mixed and incubated for 60 minutes at room temperature. The residual infectivity of the virus-serum mixture was determined in Vero (ancestral and Delta variant) or Vero TMPRSS2 (Omicron B.A.1 variant) cell lines using four wells for each dilution of serum. Neutralising antibody titre was defined as the reciprocal of the serum dilution that completely neutralised the infectivity of 100 TCID_50_of each SARS-CoV-2 strain as determined by the absence of cytopathic effect on Vero or Vero TMPRSS2 cells at day 4 as previously described.^46^ ^-^ ^48^ These studies were performed at the Physical Containment Level 3 (PC3) facility at the Peter Doherty Institute in Melbourne, Australia. 

*SARS-CoV-2 genome sequencing -* The cDNA from positive samples was used to perform genome amplification using the ARTIC_3 system (https://artic.network/ncov-2019). Amplicons were visualised on 1% agarose gels and purified with AMPure XP beads (Beckman Coulter, Indianapolis, USA). Purified amplicons (100 ng) were subjected to the Illumina DNA Prep kit (Illumina, Foster City, USA), as previously described.^49^ ^-^ ^51^ Genome sequencing was performed on the Genomic Platform at the Facultad de Ciencias, Universidad de la República Uruguay (UdelaR), using an Illumina MiniSeq (Illumina) with MiniSeqTM Mid Output Reagent Cartridge (300 cycles, paired-end reads). Adapter/quality trimming and filtering of raw data were performed with BBDuk, and reference assembly was made using Geneious Prime 2020.1.2 (https://www.geneious.com).

The complete procedure was performed in duplicate for confirmatory purposes, thus obtaining two genomic sequences for each sample.

*SARS-CoV-2 lineage classification -* Genome consensus sequences were assigned to their corresponding SARS-CoV-2 lineage according to the nomenclature system proposed by Rambaut et al.^52^

For phylogenetic analysis, two datasets were built. The first dataset comprises dog and cat sequences from the GISAID EpiCoV database^53^ ^)^ with associated metadata (Supplementary data - Table I). There are 96 and 143 complete and partial sequences from dogs and cats, respectively (up to 2022-07-21). Only complete sequences with less than 1% of unassigned base (Ns) were selected for phylogenetic analysis (number = 124) (Supplementary data - Table I). The second dataset comprised high-quality complete sequences belonging to the different circulating lineages from Uruguay, including P.6 lineage. 

The associated metadata was retrieved from GISAID (Supplementary data - Table II).

DNA alignments were performed with MAFFT.^54^ With 1,000-replicates bootstrap to support internal nodes, maximum-likelihood trees were inferred in Geneious using PhyML^55^ and visualised with the ggTree package in RStudio.

## RESULTS

*Samples and animal clinical signs -* Thirty-nine animals were sampled from 21 visited houses (24 dogs and 15 cats). The age ranges of the dogs were between 3 months and 18 years, while in the felines, it was between 8 months and 15 years. The crossbreed was the most frequently recorded breed of dog. Twenty-nine animals (19 dogs and 10 cats) belonged to households within group 1 samples, and 10 domestic animals (five dogs and five cats) belonged to households in group 2 samples (Table).

From the clinical point of view, all the animals were healthy except for one 1.5-year-old male cat corresponding to group 2, which showed symptoms of respiratory disease (epiphora, sneezing and cough), with a temperature of 38.7ºC. His general condition was good; he was not listless and had a normal appetite.

Blood biochemistry showed, in some cases, expectable alterations in the hemogram and renal function (urea and creatinine) according to the age of each animal.

*Screening by qPCR -* Nasopharyngeal and rectal swabs of 39 animals were analysed individually by real-time reverse transcription-PCR. 

A nasopharyngeal sample from a dog was positive for SARS-CoV-2 with a Ct value of 32 (Table). Unfortunately, the rectal sample for this animal was not available. The positive dog was a 3-year-old male of the Rottweiler breed living in the North Carrasco neighboUrhood in Montevideo. 

In that house lived three people; two developed symptoms in early December 2020 and were diagnosed as COVID-19 positive by qPCR on December 7, 2020. The dog sample was taken 5 days after the owner’s diagnosis date. This dog lived with a 3-year-old female dog of the Uruguayan Cimarrón breed that was SARS-CoV-2 negative.

Among the 15 cat samples, we found one SARS-CoV-2 positive case. The nasopharyngeal sample showed a Ct value of 30.8, while the rectal sample had a Ct value of 37 (Table). The positive cat was a 1-year-old male mixed breed living in the Pocitos neighbourhood in Montevideo. It lived with a 1-year-old female crossbred cat who was not positive for the presence of the virus. Only one person lived in the house who developed symptoms in December 2020 and was diagnosed as COVID-19 positive by qPCR on December 12, 2020. The cat samples were taken 5 days after the owner was diagnosed. The owner was in close contact with the animal during the disease period. 

*Serum-neutralising antibody against ancestral SARS-CoV-2 as well as Delta and Omicron variants after infection -* Sera from both the dog and the cat were collected 5 days after the SARS-CoV-2 diagnosis of their owners [on December 12, 2020 (dog) and December 17, 2020 (cat)] to determine neutralising antibodies against the ancestral Wuhan-1 like SARS-CoV-2 and the two currently circulating variants in Uruguay (Delta and Omicron B.A.1) at the moment of the sample analysis **(**https://www.gub.uy/ministerio-salud-publica/comunicacion/noticias/casos-activos-nuevas-variantes-sars-cov-2-uruguay-dia-hoy and https://www.gub.uy/ministerio-salud-publica/comunicacion/noticias/comunicado-ingreso-variante-omicron-uruguay**).** No neutralising antibodies were detected against any SARS-CoV-2 variant in the infected dog. However, antiserum from the infected cat had neutralising antibodies against the ancestral SARS-CoV-2 with a geometric mean titre (GMT) of 160. We observed cross-reactive antibodies against the Delta variant (GMT = 80) but not against the Omicron variant (GMT < 10). 

*SARS-CoV-2 genome analysis -* The nasopharyngeal samples from the dog and the cat were multiplexed, and the amplicons obtained were used for Illumina libraries construction. 

The genome assembly had 29,861 and 29,863 nt for the dog and cat, respectively. Both genomes had an average coverage depth of 1,260 × for the dog and 1,672 × for the cat, and the nucleotide consensus sequences for each species were 100% identical. 

The sequences have 15 amino acid replacements relative to the Wuhan-Hu-1 reference (MN908947.3) in nsp2 (P91L and V577F), nsp3 (P1442S), nsp4 (T492I), nsp7 (L71F), nsp12 (T225A and P323L), nsp16 (R216H), in S (D614G, Q675H, Q677H, and V1176F), in ORF3a (M260I) and N (R203K and G204R). 

Sequence data have been deposited on GenBank with accessions OM966899 and OM966900 for hCoV-19/dog/Uruguay/I_1_MO/2020 and hCoV-19/cat/Uruguay/M_1_MO/2020, respectively. 

*Classification and phylogenetic analysis -* Pangolin web application assigned the non-human variant to B.1.1.28.6 lineage, also known as P.6.

**Fig. 1:**
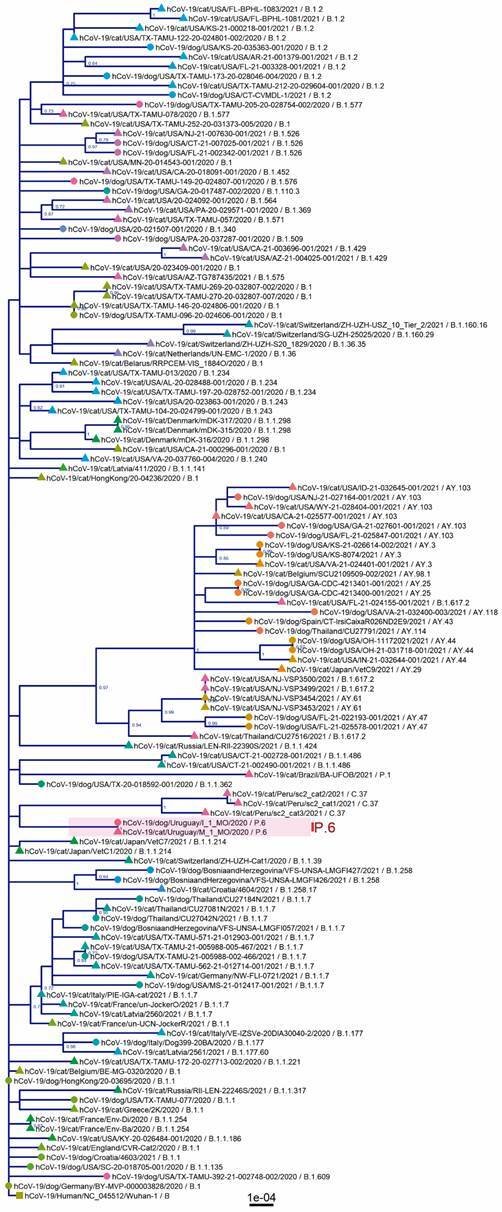
phylogenetic analysis based on 124 complete severe acute respiratory syndrome coronavirus 2 (SARS-CoV-2) genomes from domestic animals. The maximum-likelihood tree was inferred in Geneious using PhyML (GTR model) with 100-replicates bootstrap to support internal nodes. Tip labels include host, country, strain name, year and lineage. Circles represent *Canis lupus* familiaris, triangles represent *Felis catus* and the square corresponds to the humans’ reference sequence (NC_045512/Wuhan-1); tips were coloured according to the lineage.

Both P.6 sequences from the Uruguayan domestic animals were compared with dog and cat SARS-CoV-2 genomes available in the GISAID. SARS-CoV-2 variants are associated according to the lineage classification, and the phylogeny is not structured by the host (Fig. 1).

**Fig. 2:**
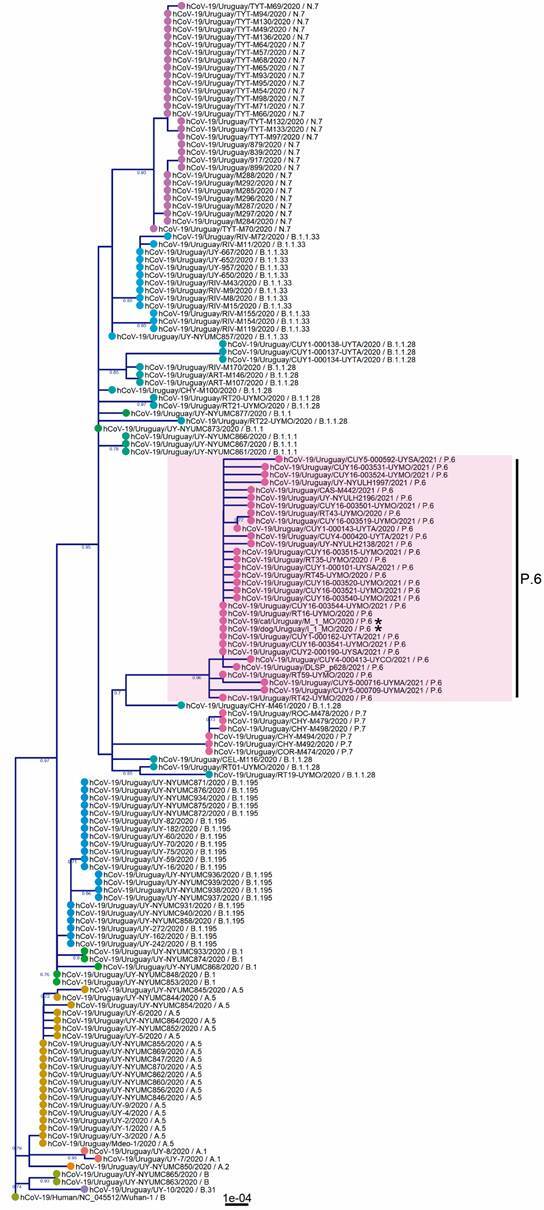
phylogenetic analysis based on 155 complete severe acute respiratory syndrome coronavirus 2 (SARS-CoV-2) genomes from Uruguay. The maximum-likelihood tree was inferred in Geneious using PhyML (GTR model) with 100-replicates bootstrap to support internal nodes. Tip labels include host, country, strain name, year and lineage. Pink shadow highlights the lineage P.6 and the asterisks, the domestic animal sequences. Tips were coloured according to the lineage.

Phylogenetic analysis with Uruguayan sequences revealed that the dog and cat genome sequences were associated with human strains belonging to the lineage P.6 (Fig. 2). Some strains of the lineage B.1.1.28 are basal for P.6. The B.1.1.28.7 or P.7 lineage appears as a sister lineage of P.6 and has related B.1.1.28 strains. These results were confirmed using all available P.6 sequences from Uruguay (Supplementary data - Figure).

## DISCUSSION

Early in the pandemic, it was noticed that SARS-CoV-2 might infect domestic cats and dogs. Accumulating evidence indicates that these and other mammals are highly susceptible to infection. Therefore, detecting SARS-CoV-2 infection in domestic animals is essential to understanding the epidemiology and identifying alternative viral transmission routes. 

The present study screened domestic animals from SARS-CoV-2-positive household cases. Animal samples were collected in the capital city of Uruguay, the region more severely affected by COVID-19 outbreaks in the country, and that offered the most convenient access to sample collection. In the sampling time frame, Uruguay was in the first pandemic wave (https://www.gub.uy/ministerio-salud-publica/comunicacion/noticias/covid-19-uruguay-del-13-marzo-13-setiembre). The frequency of COVID-19 cases started to increase steadily, reaching a daily incidence peak of 11.4 cases/100,000 people in December 2020. 

We detected the SARS-CoV-2 virus in 2/39 samples analysed. Both cases were notified to the Office International des Epizooties (OIE) (https://wahis.oie.int/#/report-info?reportId=33930), representing the first SARS-CoV-2 cases identified in domestic animals from Uruguay. Infections occurred in households where some people were SARS-CoV-2 positive for up to 2 weeks (group 1). Similar results were observed in domestic animals from other countries where the virus was detected around a week after owner infection.^25^ ^,^ ^56^ ^,^ ^57^ ^,^ ^58^

The two SARS-CoV-2 positive cases detected in domestic animals correspond to 4 and 7% samples from dogs and cats. These values do not represent the population frequency because the sample was reduced and biased (restricted to COVID-19-positive households). However, infection in domestic animals was likely underestimated in the Uruguayan pet population. Several reports have previously identified positive cases in domestic animals in the Americas, Asia and Europe.^59^ Some studies in Asia and Brazil showed higher proportions of SARS-CoV-2 positive cases in domestic animals, reaching 12 to 40%.^29^ ^,^ ^56^ A recent surveillance study in Ecuador detected a high SARS-CoV-2 positivity rate by qPCR (> 21%) in pets.^30^

Studies of natural infections and susceptibility established that the affinity of ACE2 for the S protein is higher in cats than in dogs.^26^ ^,^ ^35^ ^,^ ^60^ ^,^ ^61^ Even though the relative frequency of positive dogs in Uruguay seems higher, it is necessary to increase the sample size to confirm this trend. 

In the SARS-CoV-2-positive cat, the presence of the virus was analysed in both nasopharyngeal and rectal samples but was undetectable (Ct = 37) in the latter. This is in line with previous studies in which, in experimentally infected cats, the virus was not detected in rectal swabs from asymptomatic animals.^62^

The only available nasopharyngeal sample of the dog was positive, with a slightly higher value (Ct = 32) than the cat specimen (Ct = 30.8), suggesting a lower viral load. In dogs, experimental infection studies did not detect the virus in any tissue after being euthanized 4 days post-infection. In addition, cohoused dogs were not infected.^35^ ^,^ ^63^ The short time between the SARS-CoV-2 diagnosis of cat and dog owners and serum collection (5 days) may explain the low or undetectable titres of neutralising antibodies in the cat and the dog, respectively. Interestingly, cross-neutralising antibodies against the Delta variant (but not Omicron) were detected in the cat’s serum, which is in line with data obtained from human populations after vaccination or infection.^64^ Together, these findings suggest that the viral load and shedding undergo different decline rates in domestic animals and may eventually produce short and long-term infection, likely depending on the clinical phase or outcome of the disease and host intrinsic characteristics. There are a few longitudinal studies on pets. Hamer et al.^65^ observed viral RNA for 25 days post-initial detection in natural SARS-CoV-2-infected cats. Additionally, they found neutralising antibodies over the 2-3 months upon initial sampling. Decaro et al.^66^ showed that the SARS-CoV-2 neutralising antibodies persisted for up to 10 months in pets. However, our limited non-longitudinal sampling did not permit prevalence estimation or neutralising antibody studies in SARS-CoV-2-negative animals. In South America, SARS-CoV-2 in non-human hosts has also been notified in Argentina (dogs and domestic and wild felids), Brazil (domestic and wild animals), Chile (cats), Colombia (lion), Ecuador (domestic animals) and Peru (cats).^29^ ^,^ ^31^ ^,^ ^67^ ^-^ ^69^

Complete genome sequences data is available from Argentina (two cats and a dog of lineage B.1.1.7 and B.1.499), Brazil (one cat of lineage B.1.1.28.1 or P.1, Gamma VOC), Chile (three cats of lineage B.1.1), Colombia (one cat and a dog of lineage B.1.111 and B.1.625), Ecuador (three dogs of lineage B.1.1 and B.1.526) and Peru (three cats of lineage B.1.1.1.37 or C.37, Lambda VOI).^30^ ^-^ ^33^ ^,^ ^67^

The complete genomes obtained here belong to the P.6 lineage. This lineage emerged in Uruguay through local differentiation of the lineage B.1.1.28. The lineages B.1.1.28 and B1.1.33, with multiple introductions from the bordering country of Brazil, were predominant in the first months of the pandemic in Uruguay. During this period, the pandemic in Uruguay remained with few cases and under control. The first wave occurred at the end of 2020 with the exponential growth of cases. The P.6 lineage emerged late in November 2020 and spread throughout Uruguay, replacing the previously existing lineages, B.1.1.33 and B.1.1.28.^70^ ^,^ ^71^ The P.6 lineage emergence coincides with the exponential increase in cases in Uruguay. According to data submission at Gisaid, P.6 variants were detected between 2020-12-02 and 2021-04-26, a few days before the sample’s collection date (2020-12-12). The P.6 lineage was successful for a short period; then, it was replaced by the P.1 variant of concern.

Some mutations in the P.6 lineage acquired by convergent evolution (Q675H+Q677H) might be essential, affecting viral transmissibility with change D614G.^70^ The sequences obtained from domestic animals also retain these changes, but no unique mutations differentiate humans from domestic P.6 variants. Phylogenetic analysis with the dataset of domestic animals reveals that different lineages infect dogs and cats (Fig. 1). Furthermore, similar to lineage P.6 in Uruguay, viruses of the same lineage infect both hosts in some countries, including Argentina, Italy and the United States (Fig. 1). Thus, lineage distribution in non-human animals is geographically structured and may depend on circulating lineages in the human population, supporting infection routes from humans to dogs. In those cases where the genomic characterisation of humans and cohabiting domestic animals was possible, SARS-CoV-2 genomes were identical or shared a high identity.^23^ ^,^ ^56^ Genomic data obtained from pets and their owners also support human-to-pet transmission.^32^ ^,^ ^33^ In this study, the owners’ sequence data is unavailable, but phylogenetic analysis revealed close clustering with P.6 strains from human hosts (Fig. 2, Supplementary data - Figure). We do not have records of a possible direct epidemiological link between the households to explain the identity between both variants; the P.6 lineage is highly homogeneous, and some strains have complete nucleotide identity (100%). 

Given the evolutionary nature of coronaviruses, it becomes necessary to continue studying the role of susceptible animals in close contact with humans in the epidemiology and viral dynamics of SARS-CoV-2 infection. Therefore, regular surveillance of domestic and wild species is necessary. The genomic characterisation of these variants should be monitored to gain novel insights into the global knowledge of the evolution of SARS-CoV-2 and other related coronaviruses. Furthermore, given the susceptibility of domestic animals and other animals to SARS-CoV-2, people suspected or confirmed of COVID-19 should limit contact with animals to minimise animal infection from human sources.^29^ Additionally, animals belonging to owners infected with SARS-CoV-2 should be kept indoors in line with similar isolation recommendations for humans to prevent potential animal-to-animal spread.^11^ Overall, countries should implement One Health as a prevention and control strategy to protect both humans and animals from being infected, positively impacting the mitigation and control of disease effects and consecutively in the economy.^11^

Based on current knowledge, infected domestic animals are unlikely to play an active role in SARS-CoV-2 transmission to humans. Therefore, detecting SARS-CoV-2 does not have to provoke unnecessary fear among the public, leading to abandoning companion animals or compromising their welfare. Instead, promoting animal epidemic prevention and control strategies like Asiatic, European and North American countries is recommended based on a One Health approach.

The present work contributes to the idea that the One Health approach is desirable in controlling the current SARS-CoV-2 pandemic and delineating strategies for managing many other infectious diseases that pose public health threats.

*Impacts -* SARS-CoV-2 was detected in an asymptomatic dog and a cat during Uruguay’s exponential increase in human COVID-19 cases; complete genomes belong to the B.1.1.28.6 or P.6 SARS-CoV-2 lineage and have 100% identity with variants that infect humans; the antibodies generated by the SARS-CoV-2 infection in cats protect against B and Delta variants but not against the newer Omicron strain.
