# Supplementary material for: Detection and genome characterisation of SARS-CoV-2 P.6 lineage in dogs and cats living with Uruguayan COVID-19 patients
Source: Mem Inst Oswaldo Cruz. 2023 Jan 16;117:e220177. doi: 10.1590/0074-02760220177 (PMC9870267; doi:10.1590/0074-02760220177)
Supplement: Supplementary file 1 [file 1678-8060-mioc-117-e220177-s.pdf]

TABLE I

Dog and cat sequences with associated metadata from the GISAID's EpiCoV™ database

strain	gisaid_epi_isl	genbank_acc	date	country	pangolin_line	originating_lab
hCoV-19/dog/USA/GA-22-002465-001/2022	EPI_ISL_10656102		2022-01-01	USA	BA.1.1	Antech Diagnostics
hCoV-19/dog/Spain/MD-VISAVET-SUAT_8/2022	EPI_ISL_11580532		2022-01-01	Spain	BA.1.17	VISAVET-SUAT Complutense University of Madrid
hCoV-19/dog/Switzerland/ZH-UZH-P1841_USZ_37_T_1/2021	EPI_ISL_11583325		2021-12-08	Switzerland	AY.43.4	Clinical Laboratory, Vetsuisse Faculty, University of Zurich
hCoV-19/dog/Mexico/CPALB32021033/2020	EPI_ISL_11991713		2020-04-27	Mexico	B.1.189	ComisiÃ³n MÃ©xico-Estados Unidos (CPA-SENASICA)
hCoV-19/dog/USA/CT-CVMDL-1/2021	EPI_ISL_1241386		2021-02-12	USA	B.1.2	Connecticut Veterinary Medical Diagnostic Laboratory
hCoV-19/USA/AZ-TG1054469/2021	EPI_ISL_12543431		2021-12-03	USA	AY.103	The Translational Genomics Research Institute (TGen) North
hCoV-19/USA/AZ-TG1054531/2021	EPI_ISL_12543433		2021-11-17	USA	AY.25.1	The Translational Genomics Research Institute (TGen) North
hCoV-19/USA/AZ-TG1054539/2021	EPI_ISL_12543434		2021-11-17	USA	AY.25.1	The Translational Genomics Research Institute (TGen) North
hCoV-19/USA/AZ-TG713679/2021	EPI_ISL_12543439		2021-03-16	USA	B.1.575	The Translational Genomics Research Institute (TGen) North
hCoV-19/dog/USA/TX-TAMU-091-22-014776-001/2022	EPI_ISL_13101428		2022-04-29	USA	BA.2.3.4	Wisconsin Veterinary Diagnostic Laboratory
hCoV-19/dog/USA/TX-TAMU-21-005988-002-466/2021	EPI_ISL_1315074		2021-02-12	USA	B.1.1.7	Texas A&M College of Veterinary Medicine & Biomedical Sciences-Madison
hCoV-19/dog/Spain/CT-IrsiCaixaR009AO3B1/2021	EPI_ISL_13608277		2021-02-18	Spain	B.1.1.7	IRTA-CReSA
hCoV-19/dog/Germany/BY-MVP-000003828/2020	EPI_ISL_1750982		2020-11-24	Germany	B.1	Max von Pettenkofer Institute, Virology, LMU Munich
hCoV-19/dog/Thailand/CU27042N/2021	EPI_ISL_2628963		2021-05-04	Thailand	B.1.1.7	Department of Veterinary Public Health, Chulalongkorn University, Thailand
hCoV-19/dog/Thailand/CU27184N/2021	EPI_ISL_2663240		2021-05-19	Thailand	B.1.1.7	Department of Veterinary Public Health, Chulalongkorn University, Thailand
hCoV-19/dog/Croatia/4603/2021	EPI_ISL_2674897		2021-04	Croatia	B.1.1	Veterinarski Fakultet SveuÄ•iliÅ¡a u Zagrebu
hCoV-19/dog/USA/MS-21-012417-001/2021	EPI_ISL_2930558		2021-04-16	USA	B.1.1.7	IDEXX Reference Laboratories
hCoV-19/dog/USA/CT-21-007025-001/2021	EPI_ISL_2930560		2021-03-03	USA	B.1.526	IDEXX Reference Laboratories
hCoV-19/dog/USA/FL-21-002342-001/2021	EPI_ISL_2958983		2021-01-15	USA	B.1.526	IDEXX Reference Laboratories
hCoV-19/dog/USA/PA-20-037287-001/2020	EPI_ISL_3010051		2020-12-14	USA	B.1.509	Penn Vet University of Pennsylvania Ryan Veterinary Hospital
hCoV-19/dog/USA/KS-20-035363-001/2020	EPI_ISL_3010052		2020-12	USA	B.1.2	Kansas State Veterinary Diagnostic Laboratory
hCoV-19/dog/USA/TX-TAMU-205-20-028754-002/2020	EPI_ISL_3128553		2020-09-24	USA	B.1.577	Wisconsin Veterinary Diagnostic Laboratory
hCoV-19/dog/USA/TX-TAMU-173-20-028046-004/2020	EPI_ISL_3128555		2020-09-14	USA	B.1.2	Wisconsin Veterinary Diagnostic Laboratory
hCoV-19/dog/USA/TX-TAMU-149-20-024807-001/2020	EPI_ISL_3128557		2020-08-21	USA	B.1.576	Wisconsin Veterinary Diagnostic Laboratory
hCoV-19/dog/USA/TX-TAMU-392-21-002748-002/2020	EPI_ISL_3148877		2020-12-22	USA	B.1.609	Wisconsin Veterinary Diagnostic Laboratory
hCoV-19/dog/USA/TX-TAMU-096-20-024606-001/2020	EPI_ISL_3148880		2020-08-11	USA	B.1	Wisconsin Veterinary Diagnostic Laboratory
hCoV-19/dog/USA/20-021507-001/2020	EPI_ISL_3148884		2020-07-22	USA	B.1.340	IDEXX Reference Laboratories
hCoV-19/dog/USA/SC-20-018705-001/2020	EPI_ISL_3152898		2020-06-26	USA	B.1.1.135	Antech Diagnostics
hCoV-19/dog/USA/TX-20-018592-001/2020	EPI_ISL_3152899		2020-06-26	USA	B.1.1.362	IDEXX Reference Laboratories
hCoV-19/dog/USA/GA-20-017487-002/2020	EPI_ISL_3152901		2020-06-22	USA	B.1.110.3	University of Georgia Veterinary Diagnostic Laboratory
hCoV-19/dog/USA/KS-8074/2021	EPI_ISL_4253995		2021-09-07	USA	AY.3	Kansas State University Veterinary Health Center
hCoV-19/dog/USA/GA-CDC-4213400-001/2021	EPI_ISL_4296308		2021-07-20	USA	AY.25	Centers for Disease Control & Prevention
hCoV-19/dog/USA/GA-CDC-4213401-001/2021	EPI_ISL_4296309		2021-07-20	USA	AY.25	Centers for Disease Control & Prevention
hCoV-19/dog/HongKong/20-03695/2020	EPI_ISL_450403		2020-03-18	Hong Kong	B.1.1	School of Public Health, The University of Hong Kong
hCoV-19/dog/BosniaandHerzegovina/VFS-UNSA-LMGFI057/2021	EPI_ISL_5194358		2021-03-02	Bosnia and He	B.1.1.7	University of Sarajevo, Veterinary Faculty
hCoV-19/dog/Thailand/CU27791/2021	EPI_ISL_5315539		2021-09-14	Thailand	AY.114	Center of excellence for emerging and re-emerging diseases-Chulalongkorn
hCoV-19/dog/USA/FL-21-025847-001/2021	EPI_ISL_5761515		2021-08-25	USA	AY.103	Antech Diagnostics
hCoV-19/dog/USA/FL-21-025578-001/2021	EPI_ISL_5761517		2021-08-18	USA	AY.47	IDEXX Reference Laboratories
hCoV-19/dog/USA/FL-21-022193-001/2021	EPI_ISL_5761526		2021-07-26	USA	AY.47	Bronson Animal Disease Diagnostic Laboratory
hCoV-19/dog/USA/KS-21-026614-002/2021	EPI_ISL_5781751		2021-09-07	USA	AY.3	Kansas State Veterinary Diagnostic Laboratory
hCoV-19/dog/USA/NJ-21-027164-001/2021	EPI_ISL_5781752		2021-09-05	USA	AY.103	Antech Diagnostics
hCoV-19/dog/USA/GA-21-027601-001/2021	EPI_ISL_5781753		2021-08-26	USA	AY.103	IDEXX Reference Laboratories
hCoV-19/dog/USA/OH-OSU-0508-031718/2021	EPI_ISL_6088012		2021-09-24	USA	AY.44	The Ohio State University Department of Veterinary Preventive Medicine
hCoV-19/dog/USA/VA-21-032400-003/2021	EPI_ISL_6088079		2021-10-22	USA	AY.118	Diagnostic Virology Laboratory, National Veterinary Services Laboratories, USA
hCoV-19/dog/Spain/CT-IrsiCaixaR026ND2E9/2021	EPI_ISL_6344510		2021-07-26	Spain	AY.43	IRTA-CReSA
hCoV-19/dog/BosniaandHerzegovina/VFS-UNSA-LMGFI426/2021	EPI_ISL_6949571		2021-01-27	Bosnia and He	B.1.258	University of Sarajevo, Veterinary Faculty
hCoV-19/dog/BosniaandHerzegovina/VFS-UNSA-LMGFI427/2021	EPI_ISL_6949572		2021-03-02	Bosnia and He	B.1.258	University of Sarajevo, Veterinary Faculty
hCoV-19/dog/USA/TX-TAMU-077/2020	EPI_ISL_699508		2020-07-28	USA	B.1.1	Diagnostic Virology Laboratory, USDA National Veterinary Services Laboratories
hCoV-19/dog/Italy/Dog399-20BA/2020	EPI_ISL_730652		2020-11-04	Italy	B.1.177	University of Bari, Valenzano, Italy
hCoV-19/dog/USA/OH-OSU-0508-11172021/2021	EPI_ISL_7373930		2021-09-24	USA	AY.44	The Ohio State University Department of Veterinary Preventive Medicine
hCoV-19/dog/USA/WA-DDL21-10981-4/2021	EPI_ISL_7845317		2021-07-15	USA	AY.25	Washington Animal Disease Diagnostic Laboratory
hCoV-19/dog/USA/WA-DDL21-10981-5/2021	EPI_ISL_7845318		2021-07-15	USA	AY.25	Washington Animal Disease Diagnostic Laboratory
hCoV-19/dog/USA/MA-21-038692-001/2021	EPI_ISL_8215762		2021-12-14	USA	AY.39	Antech Diagnostics
hCoV-19/dog/USA/TX-TAMU-282-20-032807-012/2020	EPI_ISL_8317060		2020-10-22	USA	B.1.2	Wisconsin Veterinary Diagnostic Laboratory
hCoV-19/dog/Colombia/COR-U117/2021	EPI_ISL_8422346		2021-04-26	Colombia	B.1.625	Instituto de Investigaciones Biologicas del Tropico - Universidad de CÃ³rdoba
hCoV-19/dog/Switzerland/ZH-UZH-P1784_USZ22_Tier1_F1/2021	EPI_ISL_9461294		2021-11-16	Switzerland	AY.129	Clinical Laboratory, Vetsuisse Faculty, University of Zurich
hCoV-19/dog/Switzerland/ZH-UZH-P1784_USZ29_Tier1/2021	EPI_ISL_9461295		2021-11-24	Switzerland	AY.4	Clinical Laboratory, Vetsuisse Faculty, University of Zurich
hCoV-19/dog/Italy/ABR-IZSGC-319425/2021	EPI_ISL_9906009		2021-12-20	Italy	AY.23	Istituto Zooprofilattico Sperimentale dellâ Abruzzo e Molise
hCoV-19/cat/Switzerland/ZH-UZH-Cat1/2020	EPI_ISL_1005699		2020-11-16	Switzerland	B.1.1.39	Clinical Laboratory, Vetsuisse Faculty, University of Zurich
hCoV-19/cat/Spain/MD-2162/2021	EPI_ISL_10126704		2021-10-03	Spain	AY.9.2	VISAVET
hCoV-19/cat/USA/OH-21-039013-001/2021	EPI_ISL_10656103		2021-12-10	USA	AY.100	IDEXX Reference Laboratories
hCoV-19/cat/USA/CA-21-039419-001/2021	EPI_ISL_10656104		2021-12-21	USA	AY.20	IDEXX Reference Laboratories
hCoV-19/USA/IN-DSH-1/2021	EPI_ISL_11348757		2021-11-11	USA	AY.46.4	Animal Disease Diagnostic Laboratory, Purdue University
hCoV-19/cat/Spain/MD-VISAVET-SUAT_19/2022	EPI_ISL_11580576		2022-01-17	Spain	BA.1.17	VISAVET-SUAT
hCoV-19/cat/Switzerland/ZH-UZH-P1841_USZ36_T1/2021	EPI_ISL_11583324		2021-12-08	Switzerland	AY.43	Clinical Laboratory, Vetsuisse Faculty, University of Zurich
hCoV-19/cat/Switzerland/ZH-UZH-P1841_USZ38_T_2/2021	EPI_ISL_11583327		2021-12-14	Switzerland	AY.4	Clinical Laboratory, Vetsuisse Faculty, University of Zurich
hCoV-19/cat/Switzerland/ZH-UZH-P1841_USZ39_T_1/2021	EPI_ISL_11583331		2021-12-09	Switzerland	AY.43	Clinical Laboratory, Vetsuisse Faculty, University of Zurich
hCoV-19/cat/Switzerland/ZH-UZH-P1841_USZ39_T_2/2021	EPI_ISL_11583332		2021-12-09	Switzerland	AY.43	Clinical Laboratory, Vetsuisse Faculty, University of Zurich

strain	gisaid_epi_isl	genbank_acc	date	country	pangolin_line	originating_lab
hCoV-19/cat/Switzerland/ZH-UZH-P1841_USZ_41_T_3_F2/2022	EPI_ISL_11583333		2022-01-08	Switzerland	AY.42	Clinical Laboratory, Vetsuisse Faculty, University of Zurich
hCoV-19/cat/USA/FL-BPHL-1081/2021	EPI_ISL_1218882		2021-02-22	USA	B.1.2	Florida Bureau of Public Health Laboratories
hCoV-19/cat/USA/FL-BPHL-1083/2021	EPI_ISL_1218884		2021-02-22	USA	B.1.2	Florida Bureau of Public Health Laboratories
hCoV-19/USA/AZ-TG713627/2021	EPI_ISL_12543438		2021-03-16	USA	B.1.575	The Translational Genomics Research Institute (TGen) North
hCoV-19/cat/USA/TX-TAMU-21-005988-005-467/2021	EPI_ISL_1315075		2021-02-12	USA	B.1.1.7	Texas A&M College of Veterinary Medicine & Biomedical Sciences-Madison
hCoV-19/cat/Japan/VetC1/2020	EPI_ISL_1358217		2020-09	Japan	B.1.1.214	Department of Veterinary Science, National Institute of Infectious Diseases
hCoV-19/cat/Japan/VetC7/2021	EPI_ISL_1358218		2021-01	Japan	B.1.1.214	Department of Veterinary Science, National Institute of Infectious Diseases
hCoV-19/cat/USA/IN-Spaghetti/2022	EPI_ISL_13647600		2022-06-22	USA	BA.5.2.1	Animal Disease Diagnostic Laboratory, Purdue University
hCoV-19/cat/USA/AZ-TG787435/2021	EPI_ISL_1525089		2021-03-16	USA	B.1.575	Sonora Quest Laboratories
hCoV-19/cat/Belarus/RRPCEM-VIS_1884O/2020	EPI_ISL_2100634		2020-11-19	Belarus	B.1	The Laboratory of Biosafety with Pathogens Collection (RRPCEM)
hCoV-19/cat/Germany/NW-FLI-0721/2021	EPI_ISL_2365363		2021-04-09	Germany	B.1.1.7	Katzenpraxis (Cat practice) Dr. Cat
hCoV-19/cat/Switzerland/ZH-UZH-S20_1829/2020	EPI_ISL_2521766		2020-12-30	Switzerland	B.1.36.35	Clinical Laboratory, Vetsuisse Faculty, University of Zurich
hCoV-19/cat/Switzerland/SG-UZH-25025/2020	EPI_ISL_2521767		2020-12-17	Switzerland	B.1.160.29	Clinical Laboratory, Vetsuisse Faculty, University of Zurich
hCoV-19/cat/Switzerland/ZH-UZH-USZ_10_Tier_2/2021	EPI_ISL_2521769		2021-01-20	Switzerland	B.1.160.16	Clinical Laboratory, Vetsuisse Faculty, University of Zurich
hCoV-19/cat/Thailand/CU27081N/2021	EPI_ISL_2628995		2021-05-07	Thailand	B.1.1.7	Faculty of Veterinary Science, Chulalongkorn University
hCoV-19/cat/Croatia/4604/2021	EPI_ISL_2674898		2021-04	Croatia	B.1.258.17	Veterinarski Fakultet SveuÃ•iliÃ¡a u Zagrebu
hCoV-19/cat/Peru/sc2_cat1/2021	EPI_ISL_2791488		2021-03-26	Peru	C.37	Gatuario Veterinary Clinic
hCoV-19/cat/Peru/sc2_cat2/2021	EPI_ISL_2791489		2021-03-26	Peru	C.37	Gatuario Veterinary Clinic
hCoV-19/cat/Peru/sc2_cat3/2021	EPI_ISL_2791490		2021-05-02	Peru	C.37	Gatuario Veterinary Clinic
hCoV-19/cat/Italy/PIE-IGA-cat/2021	EPI_ISL_2833776		2021-03-10	Italy	B.1.1.7	Istituto Zooprofilattico Sperimentale del Piemonte, Liguria e Valle d'Aosta
hCoV-19/cat/USA/TX-TAMU-571-21-012903-001/2021	EPI_ISL_2930556		2021-04-15	USA	B.1.1.7	Wisconsin Veterinary Diagnostic Laboratory
hCoV-19/cat/USA/TX-TAMU-562-21-012714-001/2021	EPI_ISL_2930557		2021-04-15	USA	B.1.1.7	Wisconsin Veterinary Diagnostic Laboratory
hCoV-19/cat/USA/NJ-21-007630-001/2021	EPI_ISL_2930559		2021-03-06	USA	B.1.526	IDEXX Reference Laboratories
hCoV-19/cat/USA/AZ-21-004025-001/2021	EPI_ISL_2930561		2021-01-29	USA	B.1.429	IDEXX Reference Laboratories
hCoV-19/cat/USA/CA-21-003696-001/2021	EPI_ISL_2958980		2021-01-30	USA	B.1.429	IDEXX Reference Laboratories
hCoV-19/cat/USA/FL-21-003328-001/2021	EPI_ISL_2958981		2021-01-26	USA	B.1.2	IDEXX Reference Laboratories
hCoV-19/cat/USA/CT-21-002490-001/2021	EPI_ISL_2958982		2021-01-23	USA	B.1.1.486	Antech Diagnostics
hCoV-19/cat/USA/AR-21-001379-001/2021	EPI_ISL_2958984		2021-01-07	USA	B.1.2	IDEXX Reference Laboratories
hCoV-19/cat/USA/CA-21-000296-001/2020	EPI_ISL_2958985		2020-12-28	USA	B.1	IDEXX Reference Laboratories
hCoV-19/cat/USA/KS-21-000218-001/2021	EPI_ISL_2958986		2021-01-04	USA	B.1.2	Kansas State Veterinary Diagnostic Laboratory
hCoV-19/cat/USA/VA-20-037760-004/2020	EPI_ISL_3010050		2020-12-18	USA	B.1.240	Cornell Diagnostic Laboratory
hCoV-19/cat/USA/TX-TAMU-270-20-032807-007/2020	EPI_ISL_3010053		2020-10-22	USA	B.1	Wisconsin Veterinary Diagnostic Laboratory
hCoV-19/cat/USA/TX-TAMU-269-20-032807-002/2020	EPI_ISL_3010054		2020-10-22	USA	B.1	Wisconsin Veterinary Diagnostic Laboratory
hCoV-19/cat/USA/TX-TAMU-252-20-031373-005/2020	EPI_ISL_3010055		2020-10-15	USA	B.1	Wisconsin Veterinary Diagnostic Laboratory
hCoV-19/cat/Belgium/SCU2109509-002/2021	EPI_ISL_3128536		2021-07-06	Belgium	AY.98.1	Sciensano - Animal diseases
hCoV-19/cat/USA/PA-20-029571-001/2020	EPI_ISL_3128551		2020-10-02	USA	B.1.369	Antech Diagnostics
hCoV-19/cat/USA/TX-TAMU-212-20-029604-001/2020	EPI_ISL_3128552		2020-10-06	USA	B.1.2	Wisconsin Veterinary Diagnostic Laboratory
hCoV-19/cat/USA/AL-20-028488-001/2020	EPI_ISL_3128554		2020-09-25	USA	B.1.234	Thompson Bishop Sparks State Diagnostic Laboratory
hCoV-19/cat/USA/KY-20-026484-001/2020	EPI_ISL_3128556		2020-09-08	USA	B.1.1.186	Zoetis Reference Laboratories
hCoV-19/cat/USA/TX-TAMU-197-20-028752-001/2020	EPI_ISL_3128558		2020-09-22	USA	B.1.234	Wisconsin Veterinary Diagnostic Laboratory
hCoV-19/cat/USA/TX-TAMU-146-20-024806-001/2020	EPI_ISL_3128559		2020-08-21	USA	B.1	Wisconsin Veterinary Diagnostic Laboratory
hCoV-19/cat/USA/TX-TAMU-122-20-024801-002/2020	EPI_ISL_3148878		2020-08-13	USA	B.1.2	Wisconsin Veterinary Diagnostic Laboratory
hCoV-19/cat/USA/TX-TAMU-104-20-024799-001/2020	EPI_ISL_3148879		2020-08-12	USA	B.1.243	Wisconsin Veterinary Diagnostic Laboratory
hCoV-19/cat/USA/20-024092-001/2020	EPI_ISL_3148881		2020-08-17	USA	B.1.564	IDEXX Reference Laboratories
hCoV-19/cat/USA/20-023863-001/2020	EPI_ISL_3148882		2020-08-13	USA	B.1.243	IDEXX Reference Laboratories
hCoV-19/cat/USA/20-023409-001/2020	EPI_ISL_3148883		2020-08-10	USA	B.1	IDEXX Reference Laboratories
hCoV-19/cat/USA/CA-20-018091-001/2020	EPI_ISL_3152900		2020-06-25	USA	B.1.452	Antech Diagnostics
hCoV-19/cat/USA/MN-20-014543-001/2020	EPI_ISL_3152902		2020-05-20	USA	B.1	IDEXX Reference Laboratories
hCoV-19/cat/France/un-JockerO/2021	EPI_ISL_3838696		2021-03-22	France	B.1.1.7	VetAgro Sup, UniversitÃ© de Lyon
hCoV-19/cat/France/un-UCN-JockerR/2021	EPI_ISL_3857106		2021-03-22	France	B.1.1.7	VetAgro Sup, UniversitÃ© de Lyon
hCoV-19/cat/Brazil/BA-UFOB/2021	EPI_ISL_4565991		2021-05-01	Brazil	P.1	LaboratÃ³rio de Agentes Infecciosos e Vetores -LAIVE- Bahia, Barreiras, Brazil
hCoV-19/cat/France/Env-Ba/2020	EPI_ISL_483063		2020-05-14	France	B.1.1.254	unknown
hCoV-19/cat/France/Env-Di/2020	EPI_ISL_483064		2020-05-14	France	B.1.1.254	unknown
hCoV-19/cat/Belgium/BE-MG-0320/2020	EPI_ISL_487275		2020-03-11	Belgium	B.1	Department of Veterinary Pathology, University of Liege - FARAH
hCoV-19/cat/USA/CT-21-002728-001/2021	EPI_ISL_4955857		2021-01-23	USA	B.1.1.486	Antech Diagnostics
hCoV-19/cat/Japan/VetC9/2021	EPI_ISL_5104522		2021-08	Japan	AY.29	Department of Veterinary Science, National Institute of Infectious Diseases
hCoV-19/cat/Thailand/CU27516/2021	EPI_ISL_5320246		2021-07-15	Thailand	AY.30	Center of excellence for emerging and re-emerging diseases-Chulalongkorn
hCoV-19/cat/England/CVR-Cat2/2020	EPI_ISL_536400		2020-05-02	United Kingdo	B.1.1	Fareham Creek Veterinary Surgery
hCoV-19/cat/USA/VA-21-024401-001/2021	EPI_ISL_5761527		2021-08-05	USA	AY.3	IDEXX Reference Laboratories
hCoV-19/cat/USA/FL-21-024155-001/2021	EPI_ISL_5761534		2021-08-17	USA	AY.25	Bronson Animal Disease Diagnostic Laboratory
hCoV-19/cat/USA/CA-21-025577-001/2021	EPI_ISL_5761535		2021-08-17	USA	AY.103	IDEXX Reference Laboratories
hCoV-19/cat/USA/WY-21-028404-001/2021	EPI_ISL_5781756		2021-09-09	USA	AY.103	IDEXX Reference Laboratories
hCoV-19/cat/USA/IN-21-032644-001/2021	EPI_ISL_6088086		2021-10-17	USA	AY.44	IDEXX Reference Laboratories
hCoV-19/cat/USA/ID-21-032645-001/2021	EPI_ISL_6088089		2021-10-16	USA	AY.103	IDEXX Reference Laboratories
hCoV-19/cat/Latvia/2560/2021	EPI_ISL_6204713		2021-02-25	Latvia	B.1.1.7	BIOR, Institute of Food Safety Animal health and Environment
hCoV-19/cat/Latvia/2561/2021	EPI_ISL_6204714		2021-02-11	Latvia	B.1.177.60	BIOR, Institute of Food Safety Animal health and Environment
hCoV-19/cat/USA/TX-TAMU-172-20-027713-002/2020	EPI_ISL_6639377		2020-09-11	USA	B.1.1.221	Wisconsin Veterinary Diagnostic Laboratory
hCoV-19/cat/Denmark/mDK-315/2020	EPI_ISL_683164		2020-11-17	Denmark	B.1.1.298	Department of Virus and Microbiological Special Diagnostics, Denmark
hCoV-19/cat/Denmark/mDK-316/2020	EPI_ISL_683165		2020-11-17	Denmark	B.1.1.298	Department of Virus and Microbiological Special Diagnostics, Denmark
hCoV-19/cat/Denmark/mDK-317/2020	EPI_ISL_683166		2020-11-17	Denmark	B.1.1.298	Department of Virus and Microbiological Special Diagnostics, Denmark
hCoV-19/cat/USA/TX-TAMU-013/2020	EPI_ISL_699506		2020-06-28	USA	B.1.234	Diagnostic Virology Laboratory, USDA National Veterinary Services Laboratories

strain	gisaid_epi_isl	genbank_acc	date	country	pangolin_line	originating_lab
hCoV-19/cat/USA/TX-TAMU-057/2020	EPI_ISL_699507		2020-07-17	USA	B.1.571	Diagnostic Virology Laboratory, USDA National Veterinary Services Laboratories
hCoV-19/cat/USA/TX-TAMU-078/2020	EPI_ISL_699509		2020-07-29	USA	B.1.577	Diagnostic Virology Laboratory, USDA National Veterinary Services Laboratories
hCoV-19/cat/Greece/2K/2020	EPI_ISL_717979		2020-11-23	Greece	B.1.1	Laboratory of Microbiology and Infectious Diseases, Greece
hCoV-19/cat/Netherlands/UN-EMC-1/2020	EPI_ISL_722300		2020-09-08	Netherlands	B.1.36	Dutch COVID-19 response team
hCoV-19/cat/HongKong/20-04236/2020	EPI_ISL_759858		2020-03-30	Hong Kong	B.1	School of Public Health, The University of Hong Kong
hCoV-19/cat/USA/CA-21-035224-002/2021	EPI_ISL_7974436		2021-11-15	USA	AY.103	California Animal Health and Food Safety Laboratory-University of California
hCoV-19/cat/USA/IA-21-036031-001/2021	EPI_ISL_7974437		2021-11-10	USA	AY.103	Iowa State University Veterinary Diagnostic Laboratory
hCoV-19/cat/USA/TX-TAMU-250-20-031373-004/2020	EPI_ISL_7974438		2020-10-15	USA	B.1	Wisconsin Veterinary Diagnostic Laboratory
hCoV-19/cat/Latvia/411/2020	EPI_ISL_8099231		2020-12-17	Latvia	S.1	BIOR, Institute of Food Safety Animal health and Environment
hCoV-19/cat/Russia/RII-LEN-22246S/2021	EPI_ISL_811147		2021-01-06	Russia	B.1.1.317	WHO National Influenza Centre Russian Federation
hCoV-19/cat/USA/OH-21-037782-001/2021	EPI_ISL_8145732		2021-12-10	USA	AY.25.1	Antech Diagnostics
hCoV-19/cat/USA/NJ-VSP3509/2021	EPI_ISL_8599342		2021-09-22	USA	AY.3	Lennon
hCoV-19/cat/USA/NJ-VSP3510/2021	EPI_ISL_8599343		2021-09-29	USA	AY.3	Lennon
hCoV-19/cat/Russia/LEN-RII-22390S/2021	EPI_ISL_873040		2021-01-08	Russia	B.1.1.424	WHO National Influenza Centre Russian Federation
hCoV-19/cat/USA/WA-21-032634-002s/2021	EPI_ISL_8897004		2021-04-23	USA	B.1.2	Wahington Animal Disease Diagnostic Laboratory
hCoV-19/cat/Switzerland/ZH-UZH-P1784_USZ22_Tier2/2021	EPI_ISL_9461296		2021-11-16	Switzerland	AY.129	Clinical Laboratory, Vetsuisse Faculty, University of Zurich
hCoV-19/cat/Italy/VE-IZSVe-20DIA30040-2/2020	EPI_ISL_962892		2020-11-27	Italy	B.1.177	ULSS 03 Venezia
hCoV-19/cat/USA/UT-CDC-4168047-001/2020	EPI_ISL_9936845		2020-10-02	USA	B.1	One Health Office, Centers for Disease Control and Prevention
hCoV-19/cat/USA/UT-CDC-4159739-001/2020	EPI_ISL_9936852		2020-08-26	USA	B.1	One Health Office, Centers for Disease Control and Prevention
hCoV-19/Human/NC_045512/Wuhan-1	Wuhan-1	NC_045512	13/1/2020	China	B	
hCoV-19/dog/Uruguay/I_1_MO/2020		OM966899		Uruguay	P.6	Genetica, UdelaR
hCoV-19/cat/Uruguay/M_1_MO/2020		OM966900		Uruguay	P.6	Genetica, UdelaR

TABLE II

Uruguayan sequences with associated metadata from the GISAID's EpiCoV™ database

gisaid_epi_isl	date	ngolin_line	originating_lab
EPI_ISL_2754095	2020-12-26	P.7	Sanatorio Americano
EPI_ISL_2754096	2020-12-21	B.1.1.28	Sanatorio Americano
EPI_ISL_2754115	2020-12-21	B.1.1.28	Sanatorio Americano
EPI_ISL_2754129	2020-12-13	P.7	Sanatorio Americano
EPI_ISL_2754130	2020-12-10	B.1.1.28	Sanatorio Americano
EPI_ISL_2754131	2020-12-13	B.1.1.33	Sanatorio Americano
EPI_ISL_2754132	2020-12-22	P.7	Sanatorio Americano
EPI_ISL_2754139	2020-12-14	P.7	Sanatorio Americano
EPI_ISL_2754195	2020-12-20	P.7	Sanatorio Americano
EPI_ISL_2754203	2020-12-14	P.7	Sanatorio Americano
EPI_ISL_2754310	2020-12-12	P.7	Sanatorio Americano
EPI_ISL_2754311	2020-12-10	P.7	Sanatorio Americano
EPI_ISL_2754312	2020-12-12	B.1.1.33	Sanatorio Americano
EPI_ISL_2754313	2020-11-27	P.7	CURE
EPI_ISL_2754314	2020-11-30	P.7	CURE
EPI_ISL_2754315	2020-12-03	P.7	CURE
EPI_ISL_2754316	2020-12-03	P.7	CURE
EPI_ISL_2754317	2020-11-25	P.7	CURE
EPI_ISL_2754318	2020-12-07	P.7	CURE
EPI_ISL_2754370	2020-12-07	P.7	CURE
EPI_ISL_2754398	2020-12-04	P.7	CURE
EPI_ISL_2965558	2020-12-31	P.6	Hospital Español
EPI_ISL_2965561	2020-12-30	P.6	Hospital Español
EPI_ISL_2965562	2020-12-18	P.6	Hospital Español
EPI_ISL_2965563	2020-12-02	P.6	Hospital Español
EPI_ISL_2965568	2020-12-29	P.6	Hospital Español
EPI_ISL_2965569	2020-12-30	P.6	Hospital Español
EPI_ISL_2965581	2020-10-05	B.1.1.28	Hospital Español
EPI_ISL_2965583	2020-08-07	B.1.1.28	Hospital Español
EPI_ISL_2965584	2020-09-14	B.1.1.28	Hospital Español
EPI_ISL_2965585	2020-10-05	B.1.1.28	Hospital Español
EPI_ISL_2965586	2020-09-25	B.1.1.28	Hospital Español
EPI_ISL_3089977	2020-12-08	P.6	CENUR, Litoral Norte
EPI_ISL_3090038	2020-11-16	B.1.1.28	CENUR, Litoral Norte
EPI_ISL_3090039	2020-11-16	B.1.1.28	CENUR, Litoral Norte
EPI_ISL_3090040	2020-11-23	B.1.1.28	CENUR, Litoral Norte
EPI_ISL_3696840	2020-09-22	N.7	Facultad de Ciencias & DLSP
EPI_ISL_3696841	2020-09-22	N.7	Facultad de Ciencias & DLSP
EPI_ISL_3696842	2020-09-16	N.7	Facultad de Ciencias & DLSP
EPI_ISL_3696843	2020-09-18	N.7	Facultad de Ciencias & DLSP
EPI_ISL_426476	2020-03-16	A.5	Institut Pasteur Montevideo
EPI_ISL_426477	2020-03-16	A.5	Institut Pasteur Montevideo
EPI_ISL_426478	2020-03-17	A.5	Institut Pasteur Montevideo
EPI_ISL_426479	2020-03-17	A.5	Institut Pasteur Montevideo

gisaid_epi_isl	date	ngolin_line	originating_lab
EPI_ISL_426480	2020-03-17	A.5	Institut Pasteur Montevideo
EPI_ISL_426481	2020-03-17	A.5	Institut Pasteur Montevideo
EPI_ISL_426482	2020-03-17	A.1	Institut Pasteur Montevideo
EPI_ISL_426583	2020-03-17	A.1	Institut Pasteur Monteivdeo
EPI_ISL_426584	2020-03-19	A.5	Institut Pasteur Montevideo
EPI_ISL_429257	2020-03-19	B.31	Institut Pasteur Montevideo
EPI_ISL_444493	2020-03-13	A.5	DLSP, MSP
EPI_ISL_457940	2020-03-17	A.5	Asociación Española
EPI_ISL_457941	2020-03-17	A.5	Asociación Española
EPI_ISL_457942	2020-03-17	A.5	Asociación Española
EPI_ISL_457943	2020-03-17	A.5	Asociación Española
EPI_ISL_457944	2020-03-17	B.1	Asociación Española
EPI_ISL_457945	2020-03-17	A.2	Asociación Española
EPI_ISL_457946	2020-03-17	A.2	Asociación Española
EPI_ISL_457947	2020-03-17	A.5	Asociación Española
EPI_ISL_457948	2020-03-17	A.5	Asociación Española
EPI_ISL_457949	2020-03-17	B.1	Asociación Española
EPI_ISL_457950	2020-03-21	A.5	Asociación Española
EPI_ISL_457951	2020-03-21	A.5	Asociación Española
EPI_ISL_457952	2020-03-21	A.5	Asociación Española
EPI_ISL_457953	2020-03-23	B.1.1.33	Asociación Española
EPI_ISL_457954	2020-03-23	B.1.195	Asociación Española
EPI_ISL_457955	2020-03-24	B.1.1.1	Asociación Española
EPI_ISL_457956	2020-03-25	A.5	Asociación Española
EPI_ISL_457957	2020-03-25	B.1.1.1	Asociación Española
EPI_ISL_457958	2020-03-25	A.5	Asociación Española
EPI_ISL_457959	2020-03-26	B	Asociación Española
EPI_ISL_457960	2020-03-28	A.5	Asociación Española
EPI_ISL_457961	2020-03-28	B	Asociación Española
EPI_ISL_457962	2020-03-30	B.1.1.1	Asociación Española
EPI_ISL_457963	2020-03-28	B.1.1.1	Asociación Española
EPI_ISL_457964	2020-04-01	B.1	Asociación Española
EPI_ISL_457965	2020-04-03	A.5	Asociación Española
EPI_ISL_457966	2020-04-06	A.5	Asociación Española
EPI_ISL_457967	2020-04-09	B.1.195	Asociación Española
EPI_ISL_457968	2020-04-11	B.1.195	Asociación Española
EPI_ISL_457969	2020-04-14	B.1.1	Asociación Española
EPI_ISL_457970	2020-04-14	B.1	Asociación Española
EPI_ISL_457971	2020-04-19	B.1.195	Asociación Española
EPI_ISL_457972	2020-04-21	B.1.195	Asociación Española
EPI_ISL_457973	2020-04-22	B.1.1	Asociación Española
EPI_ISL_480331	2020-04-25	B.1.195	Institut Pasteur de Montevideo
EPI_ISL_480332	2020-04-07	B.1.195	Institut Pasteur de Montevideo
EPI_ISL_480333	2020-04-07	B.1.195	Institut Pasteur de Montevideo
EPI_ISL_480334	2020-04-07	B.1.195	Institut Pasteur de Montevideo
EPI_ISL_480335	2020-04-07	B.1.195	Institut Pasteur de Montevideo

gisaid_epi_isl	date	ngolin_line	originating_lab
EPI_ISL_480336	2020-04-25	B.1.195	Institut Pasteur de Montevideo
EPI_ISL_480337	2020-04-25	B.1.195	Institut Pasteur de Montevideo
EPI_ISL_480338	2020-05-25	B.1.1.33	Institut Pasteur de Montevideo
EPI_ISL_480339	2020-05-25	B.1.1.33	Institut Pasteur de Montevideo
EPI_ISL_480340	2020-05-25	B.1.1.33	Institut Pasteur de Montevideo
EPI_ISL_480341	2020-05-25	B.1.1.33	Institut Pasteur de Montevideo
EPI_ISL_480342	2020-04-25	B.1.195	Institut Pasteur de Montevideo
EPI_ISL_480343	2020-04-25	B.1.195	Institut Pasteur de Montevideo
EPI_ISL_480344	2020-04-07	B.1.195	Institut Pasteur de Montevideo
EPI_ISL_480345	2020-04-13	A.5	Institut Pasteur de Montevideo
EPI_ISL_480346	2020-05-25	B.1.1.33	Institut Pasteur de Montevideo
EPI_ISL_480347	2020-05-20	B.1.195	Institut Pasteur de Montevideo
EPI_ISL_480348	2020-05-20	B.1.195	Institut Pasteur de Montevideo
EPI_ISL_480428	2020-03-26	A.5	Asociación Española
EPI_ISL_480429	2020-04-10	B.1.195	Asociación Española
EPI_ISL_480431	2020-04-25	B.1	Asociación Española
EPI_ISL_480432	2020-05-07	B.1.195	Asociación Española
EPI_ISL_480433	2020-05-16	B.1.195	Asociación Española
EPI_ISL_480434	2020-05-19	B.1.195	Asociación Española
EPI_ISL_480435	2020-05-19	B.1.195	Asociación Española
EPI_ISL_480436	2020-05-26	B.1.195	Asociación Española
EPI_ISL_480437	2020-05-26	B.1.195	Asociación Española
EPI_ISL_480438	2020-03-18	B.1.195	Asociación Española
EPI_ISL_540430	2020-05-08	None	Institut Pasteur de Montevideo
EPI_ISL_747615	2020-05-05	B.1.1.33	IIBCE
EPI_ISL_748138	2020-05-21	B.1.1.33	IIBCE
EPI_ISL_748139	2020-05-21	B.1.1.33	IIBCE
EPI_ISL_748140	2020-05-21	B.1.1.33	IIBCE
EPI_ISL_748141	2020-05-21	B.1.1.33	IIBCE
EPI_ISL_748142	2020-05-21	B.1.1.33	IIBCE
EPI_ISL_748143	2020-05-21	B.1.1.33	IIBCE
EPI_ISL_748144	2020-05-21	B.1.1.33	IIBCE
EPI_ISL_748145	2020-05-21	B.1.1.33	IIBCE
EPI_ISL_748667	2020-05-21	B.1.1.33	Sanatorio Americano
EPI_ISL_749036	2020-05-28	B.1.1.33	Sanatorio Americano
EPI_ISL_749148	2020-06-18	N.7	Sanatorio Americano
EPI_ISL_749149	2020-06-18	N.7	Sanatorio Americano
EPI_ISL_749150	2020-06-18	N.7	Sanatorio Americano
EPI_ISL_749151	2020-06-18	N.7	Sanatorio Americano
EPI_ISL_749152	2020-06-19	N.7	Sanatorio Americano
EPI_ISL_749153	2020-06-20	N.7	Sanatorio Americano
EPI_ISL_749154	2020-06-20	N.7	Sanatorio Americano
EPI_ISL_749155	2020-06-21	N.7	Sanatorio Americano
EPI_ISL_749238	2020-06-21	N.7	Sanatorio Americano
EPI_ISL_749474	2020-06-21	N.7	Sanatorio Americano

gisaid_epi_isl	date	ngolin_line	originating_lab
EPI_ISL_749706	2020-06-21	N.7	Sanatorio Americano
EPI_ISL_749906	2020-06-21	N.7	Sanatorio Americano
EPI_ISL_750108	2020-06-21	N.7	Sanatorio Americano
EPI_ISL_750161	2020-06-21	N.7	Sanatorio Americano
EPI_ISL_750162	2020-06-22	N.7	Sanatorio Americano
EPI_ISL_750163	2020-06-05	B.1.1.33	DILAVE/MGAP-INIA-UdelaR
EPI_ISL_750164	2020-06-05	B.1.1.33	DILAVE/MGAP-INIA-UdelaR
EPI_ISL_750165	2020-06-02	B.1.1.33	CENUR Litoral Norte
EPI_ISL_750166	2020-06-21	N.7	Sanatorio Americano
EPI_ISL_750168	2020-06-24	N.7	Sanatorio Americano
EPI_ISL_750169	2020-06-25	N.7	Sanatorio Americano
EPI_ISL_750170	2020-06-25	N.7	Sanatorio Americano
EPI_ISL_750171	2020-06-26	N.7	Sanatorio Americano
EPI_ISL_750172	2020-06-26	N.7	Sanatorio Americano
EPI_ISL_750173	2020-06-27	N.7	Sanatorio Americano
EPI_ISL_750174	2020-06-27	N.7	Sanatorio Americano
EPI_ISL_750175	2020-07-16	B.1.1.28	CENUR Este-Sede Rocha-UdelaR
EPI_ISL_750176	2020-07-08	B.1.1.28	Sanatorio Americano
EPI_ISL_750177	2020-07-17	B.1.1.28	Sanatorio Americano
EPI_ISL_750178	2020-07-19	B.1.1.33	Sanatorio Americano
EPI_ISL_750179	2020-06-30	N.7	Sanatorio Americano
EPI_ISL_750256	2020-07-01	N.7	Sanatorio Americano
EPI_ISL_750430	2020-07-01	N.7	Sanatorio Americano
EPI_ISL_750820	2020-07-02	N.7	Sanatorio Americano
EPI_ISL_751011	2020-07-02	N.7	Sanatorio Americano
EPI_ISL_751184	2020-07-06	B.1.1.28	CENUR Litoral Norte
EPI_ISL_751185	2020-07-20	B.1.1.28	CENUR Litoral Norte
EPI_ISL_751186	2020-07-22	B.1.1.28	CENUR Litoral Norte
EPI_ISL_751187	2020-07-21	B.1.1.33	CENUR Litoral Norte
EPI_ISL_751188	2020-07-21	B.1.1.33	CENUR Litoral Norte
EPI_ISL_751189	2020-07-23	B.1.1.28	CENUR Litoral Norte
EPI_ISL_751190	2020-07-26	B.1.1.28	CENUR Litoral Norte
EPI_ISL_751201	2020-07-20	B.1.1.28	DILAVE/MGAP-INIA-UdelaR
EPI_ISL_936380	2020-07-14	N.7	Genetica y Virologia, Facultad de Ciencias
EPI_ISL_936381	2020-07-14	N.7	Genetica y Virologia, Facultad de Ciencias
EPI_ISL_936382	2020-07-14	N.7	Genetica y Virologia, Facultad de Ciencias
EPI_ISL_936383	2020-07-14	N.7	Genetica y Virologia, Facultad de Ciencias
EPI_ISL_936384	2020-07-14	N.7	Genetica y Virologia, Facultad de Ciencias
EPI_ISL_936385	2020-07-14	N.7	Genetica y Virologia, Facultad de Ciencias
EPI_ISL_936386	2020-07-14	N.7	Genetica y Virologia, Facultad de Ciencias
Wuhan-1	2020-01-13	B	NA
dog/Uruguay	2020-12-07	P.6	NA
cat/Uruguay	2020-12-12	P.6	NA
EPI_ISL_2965563	2020-12-02	P.6	Hospital Español
EPI_ISL_3089977	2020-12-08	P.6	CENUR, Litoral Norte

gisaid_epi_isl	date	ngolin_line	originating_lab
EPI_ISL_2965562	2020-12-18	P.6	Hospital Español
EPI_ISL_7685133	2020-12-24	P.6	Laboratoire de santé publique du Québec
EPI_ISL_2965568	2020-12-29	P.6	Hospital Español
EPI_ISL_2965561	2020-12-30	P.6	Hospital Español
EPI_ISL_2965558	2020-12-31	P.6	Hospital Español
EPI_ISL_2965560	2021-01-02	P.6	Hospital Español
EPI_ISL_2965559	2021-01-03	P.6	Hospital Español
EPI_ISL_3089978	2021-01-03	P.6	CENUR, Litoral Norte
EPI_ISL_2964381	2021-01-06	P.6	DILAVE/MGAP-INIA-UdelaR
EPI_ISL_3089979	2021-01-07	P.6	CENUR, Litoral Norte
EPI_ISL_2964705	2021-01-10	P.6	Hospital Español
EPI_ISL_3089982	2021-01-11	P.6	CENUR, Litoral Norte
EPI_ISL_2754073	2021-01-12	P.6	Sanatorio Americano
EPI_ISL_3089983	2021-01-12	P.6	CENUR, Litoral Norte
EPI_ISL_2965557	2021-01-13	P.6	Hospital Español
EPI_ISL_2963518	2021-01-14	P.6	Hospital Español
EPI_ISL_3089984	2021-01-14	P.6	CENUR, Litoral Norte
EPI_ISL_2965564	2021-01-16	P.6	Hospital Español
EPI_ISL_2754030	2021-01-17	P.6	Sanatorio Americano
EPI_ISL_2754031	2021-01-17	P.6	Sanatorio Americano
EPI_ISL_2754032	2021-01-17	P.6	Sanatorio Americano
EPI_ISL_2964383	2021-01-18	P.6	DILAVE/MGAP-INIA-UdelaR
EPI_ISL_3089985	2021-01-19	P.6	CENUR, Litoral Norte
EPI_ISL_3089986	2021-01-19	P.6	CENUR, Litoral Norte
EPI_ISL_3089980	2021-01-20	P.6	CENUR, Litoral Norte
EPI_ISL_3089987	2021-01-20	P.6	CENUR, Litoral Norte
EPI_ISL_3089988	2021-01-20	P.6	CENUR, Litoral Norte
EPI_ISL_3089981	2021-01-21	P.6	CENUR, Litoral Norte
EPI_ISL_11799613	2021-01-22	P.6	DLSP,. MSP
EPI_ISL_1213397	2021-01-22	P.6	Laboratório HLA/UERJ
EPI_ISL_3089989	2021-01-22	P.6	CENUR, Litoral Norte
EPI_ISL_3090035	2021-01-26	P.6	Institut Pasteur Montevideo
EPI_ISL_3090032	2021-01-27	P.6	Institut Pasteur Montevideo
EPI_ISL_3090033	2021-01-27	P.6	Institut Pasteur Montevideo
EPI_ISL_3090029	2021-01-28	P.6	Institut Pasteur Montevideo
EPI_ISL_3090030	2021-01-28	P.6	Institut Pasteur Montevideo
EPI_ISL_3090031	2021-01-28	P.6	Institut Pasteur Montevideo
EPI_ISL_3090026	2021-01-29	P.6	Institut Pasteur Montevideo
EPI_ISL_3090027	2021-01-29	P.6	Institut Pasteur Montevideo
EPI_ISL_3090028	2021-01-29	P.6	Institut Pasteur Montevideo
EPI_ISL_3090025	2021-02-01	P.6	Institut Pasteur Montevideo
EPI_ISL_11799616	2021-02-02	P.6	DLSP, MSP
EPI_ISL_3090022	2021-02-02	P.6	Institut Pasteur Montevideo
EPI_ISL_3090023	2021-02-02	P.6	Institut Pasteur Montevideo
EPI_ISL_3090024	2021-02-02	P.6	Institut Pasteur Montevideo

gisaid_epi_isl	date	ngolin_line	originating_lab
EPI_ISL_2427615	2021-02-03	P.6	Médica Uruguaya
EPI_ISL_2427654	2021-02-03	P.6	Médica Uruguaya
EPI_ISL_2427664	2021-02-03	P.6	Médica Uruguaya
EPI_ISL_2427691	2021-02-03	P.6	Médica Uruguaya
EPI_ISL_3090019	2021-02-03	P.6	Institut Pasteur Montevideo
EPI_ISL_3090020	2021-02-03	P.6	Institut Pasteur Montevideo
EPI_ISL_3090021	2021-02-03	P.6	Institut Pasteur Montevideo
EPI_ISL_11799580	2021-02-04	P.6	DLSP, MSP
EPI_ISL_2427617	2021-02-04	P.6	Médica Uruguaya
EPI_ISL_2427632	2021-02-04	P.6	Médica Uruguaya
EPI_ISL_2427672	2021-02-04	P.6	Médica Uruguaya
EPI_ISL_2427745	2021-02-04	P.6	Médica Uruguaya
EPI_ISL_3089996	2021-02-04	P.6	Hospital Español
EPI_ISL_2427633	2021-02-05	P.6	Médica Uruguaya
EPI_ISL_2427641	2021-02-05	P.6	Médica Uruguaya
EPI_ISL_2427647	2021-02-05	P.6	Médica Uruguaya
EPI_ISL_2427671	2021-02-05	P.6	Médica Uruguaya
EPI_ISL_3089990	2021-02-05	P.6	CENUR, Litoral Norte
EPI_ISL_3090014	2021-02-05	P.6	Institut Pasteur Montevideo
EPI_ISL_3090015	2021-02-05	P.6	Institut Pasteur Montevideo
EPI_ISL_3090016	2021-02-05	P.6	Institut Pasteur Montevideo
EPI_ISL_2427623	2021-02-06	P.6	Médica Uruguaya
EPI_ISL_2427625	2021-02-06	P.6	Médica Uruguaya
EPI_ISL_2427631	2021-02-06	P.6	Médica Uruguaya
EPI_ISL_2427640	2021-02-06	P.6	Médica Uruguaya
EPI_ISL_2427656	2021-02-06	P.6	Médica Uruguaya
EPI_ISL_3089991	2021-02-08	P.6	CENUR, Litoral Norte
EPI_ISL_3089993	2021-02-08	P.6	CENUR, Litoral Norte
EPI_ISL_3090011	2021-02-08	P.6	Institut Pasteur Montevideo
EPI_ISL_3090012	2021-02-08	P.6	Institut Pasteur Montevideo
EPI_ISL_3090013	2021-02-08	P.6	Institut Pasteur Montevideo
EPI_ISL_3089992	2021-02-09	P.6	CENUR, Litoral Norte
EPI_ISL_3089994	2021-02-10	P.6	Hospital Español
EPI_ISL_3089995	2021-02-10	P.6	Hospital Español
EPI_ISL_3090007	2021-02-10	P.6	Institut Pasteur Montevideo
EPI_ISL_3090008	2021-02-10	P.6	Institut Pasteur Montevideo
EPI_ISL_3090009	2021-02-10	P.6	Institut Pasteur Montevideo
EPI_ISL_3090010	2021-02-10	P.6	Institut Pasteur Montevideo
EPI_ISL_3090017	2021-02-10	P.6	Institut Pasteur Montevideo
EPI_ISL_3090018	2021-02-10	P.6	Institut Pasteur Montevideo
EPI_ISL_3090034	2021-02-10	P.6	Institut Pasteur Montevideo
EPI_ISL_2427609	2021-02-12	P.6	Médica Uruguaya
EPI_ISL_2427661	2021-02-12	P.6	Médica Uruguaya
EPI_ISL_3090006	2021-02-12	P.6	Institut Pasteur Montevideo
EPI_ISL_2427644	2021-02-14	P.6	Médica Uruguaya

gisaid_epi_isl	date	ngolin_line	originating_lab
EPI_ISL_2427737	2021-02-14	P.6	Médica Uruguaya
EPI_ISL_3090004	2021-02-15	P.6	Institut Pasteur Montevideo
EPI_ISL_3090005	2021-02-15	P.6	Institut Pasteur Montevideo
EPI_ISL_2427607	2021-02-16	P.6	Médica Uruguaya
EPI_ISL_2427626	2021-02-16	P.6	Médica Uruguaya
EPI_ISL_2427650	2021-02-16	P.6	Médica Uruguaya
EPI_ISL_2427658	2021-02-16	P.6	Médica Uruguaya
EPI_ISL_2427679	2021-02-16	P.6	Médica Uruguaya
EPI_ISL_3090003	2021-02-16	P.6	Institut Pasteur Montevideo
EPI_ISL_11799611	2021-02-17	P.6	DLSP, MSP
EPI_ISL_3090000	2021-02-17	P.6	Institut Pasteur Montevideo
EPI_ISL_3090001	2021-02-17	P.6	Institut Pasteur Montevideo
EPI_ISL_3090002	2021-02-17	P.6	Institut Pasteur Montevideo
EPI_ISL_2427613	2021-02-18	P.6	Médica Uruguaya
EPI_ISL_2427646	2021-02-18	P.6	Médica Uruguaya
EPI_ISL_2427678	2021-02-18	P.6	Médica Uruguaya
EPI_ISL_3089999	2021-02-18	P.6	Institut Pasteur Montevideo
EPI_ISL_3090036	2021-02-18	P.6	Institut Pasteur Montevideo
EPI_ISL_3090037	2021-02-18	P.6	Institut Pasteur Montevideo
EPI_ISL_3089997	2021-02-19	P.6	Institut Pasteur Montevideo
EPI_ISL_3089998	2021-02-19	P.6	Institut Pasteur Montevideo
EPI_ISL_2427680	2021-02-24	P.6	Médica Uruguaya
EPI_ISL_2427723	2021-02-24	P.6	Médica Uruguaya
EPI_ISL_3089975	2021-02-24	P.6	CENUR, Litoral Norte
EPI_ISL_1939070	2021-02-25	P.6	Universidad de León
EPI_ISL_2427535	2021-02-25	P.6	Médica Uruguaya
EPI_ISL_2427543	2021-02-25	P.6	Médica Uruguaya
EPI_ISL_2427551	2021-02-25	P.6	Médica Uruguaya
EPI_ISL_2427685	2021-02-25	P.6	Médica Uruguaya
EPI_ISL_2755057	2021-02-25	P.6	CURE
EPI_ISL_2963519	2021-02-25	P.6	CENUR, Litoral Norte
EPI_ISL_2427605	2021-02-26	P.6	Médica Uruguaya
EPI_ISL_2427608	2021-02-26	P.6	Médica Uruguaya
EPI_ISL_2427616	2021-02-26	P.6	Médica Uruguaya
EPI_ISL_2427621	2021-02-26	P.6	Médica Uruguaya
EPI_ISL_2427624	2021-02-26	P.6	Médica Uruguaya
EPI_ISL_2427630	2021-02-26	P.6	Médica Uruguaya
EPI_ISL_2427635	2021-02-26	P.6	Médica Uruguaya
EPI_ISL_2427637	2021-02-26	P.6	Médica Uruguaya
EPI_ISL_2427645	2021-02-26	P.6	Médica Uruguaya
EPI_ISL_2427660	2021-02-26	P.6	Médica Uruguaya
EPI_ISL_2427675	2021-02-26	P.6	Médica Uruguaya
EPI_ISL_2427686	2021-02-26	P.6	Médica Uruguaya
EPI_ISL_2427699	2021-02-26	P.6	Médica Uruguaya
EPI_ISL_3089976	2021-02-26	P.6	CENUR, Litoral Norte

gisaid_epi_isl	date	ngolin_line	originating_lab
EPI_ISL_2427568	2021-02-27	P.6	Médica Uruguaya
EPI_ISL_2427603	2021-02-27	P.6	Médica Uruguaya
EPI_ISL_2427619	2021-02-27	P.6	Médica Uruguaya
EPI_ISL_2427627	2021-02-27	P.6	Médica Uruguaya
EPI_ISL_2427629	2021-02-27	P.6	Médica Uruguaya
EPI_ISL_2427649	2021-02-27	P.6	Médica Uruguaya
EPI_ISL_2427655	2021-02-27	P.6	Médica Uruguaya
EPI_ISL_2427670	2021-02-27	P.6	Médica Uruguaya
EPI_ISL_2427662	2021-02-28	P.6	Médica Uruguaya
EPI_ISL_2517017	2021-03-01	P.6	Washington University of St. Louis
EPI_ISL_2427636	2021-03-02	P.6	Médica Uruguaya
EPI_ISL_2427651	2021-03-02	P.6	Médica Uruguaya
EPI_ISL_2964004	2021-03-02	P.6	CENUR, Litoral Norte
EPI_ISL_2427542	2021-03-03	P.6	Médica Uruguaya
EPI_ISL_2427550	2021-03-03	P.6	Médica Uruguaya
EPI_ISL_2427558	2021-03-03	P.6	Médica Uruguaya
EPI_ISL_2427564	2021-03-03	P.6	Médica Uruguaya
EPI_ISL_2427571	2021-03-03	P.6	Médica Uruguaya
EPI_ISL_2427577	2021-03-03	P.6	Médica Uruguaya
EPI_ISL_2427579	2021-03-03	P.6	Médica Uruguaya
EPI_ISL_2427585	2021-03-03	P.6	Médica Uruguaya
EPI_ISL_2427587	2021-03-03	P.6	Médica Uruguaya
EPI_ISL_2427593	2021-03-03	P.6	Médica Uruguaya
EPI_ISL_2427511	2021-03-04	P.6	Médica Uruguaya
EPI_ISL_2427592	2021-03-04	P.6	Médica Uruguaya
EPI_ISL_2963634	2021-03-04	P.6	Sanatorio Americano
EPI_ISL_2964380	2021-03-04	P.6	CENUR, Litoral Norte
EPI_ISL_2964382	2021-03-04	P.6	Sanatorio Americano
EPI_ISL_2964384	2021-03-04	P.6	Sanatorio Americano
EPI_ISL_2427516	2021-03-06	P.6	Médica Uruguaya
EPI_ISL_2427524	2021-03-06	P.6	Médica Uruguaya
EPI_ISL_2427532	2021-03-06	P.6	Médica Uruguaya
EPI_ISL_2427540	2021-03-06	P.6	Médica Uruguaya
EPI_ISL_11799617	2021-03-11	P.6	DLSP, MSP
EPI_ISL_2964385	2021-03-12	P.6	Sanatorio Americano
EPI_ISL_2964223	2021-03-13	P.6	Sanatorio Americano
EPI_ISL_2427559	2021-03-17	P.6	Médica Uruguaya
EPI_ISL_11799612	2021-03-18	P.6	DLSP, MSP
EPI_ISL_2427546	2021-03-20	P.6	Médica Uruguaya
EPI_ISL_2427539	2021-03-21	P.6	Médica Uruguaya
EPI_ISL_2427555	2021-03-21	P.6	Médica Uruguaya
EPI_ISL_1520165	2021-03-23	P.6	Northwestern Medicine
EPI_ISL_2427509	2021-03-25	P.6	Médica Uruguaya
EPI_ISL_2427517	2021-03-25	P.6	Médica Uruguaya
EPI_ISL_2427557	2021-03-25	P.6	Médica Uruguaya

gisaid_epi_isl	date	ngolin_line	originating_lab
EPI_ISL_2427578	2021-03-25	P.6	Médica Uruguaya
EPI_ISL_2964387	2021-03-28	P.6	Sanatorio Americano
EPI_ISL_2964388	2021-03-28	P.6	Sanatorio Americano
EPI_ISL_11799614	2021-03-29	P.6	DLSP, MSP
EPI_ISL_11799615	2021-03-29	P.6	DLSP, MSP
EPI_ISL_2427520	2021-03-29	P.6	Médica Uruguaya
EPI_ISL_2427528	2021-03-29	P.6	Médica Uruguaya
EPI_ISL_2964558	2021-03-29	P.6	Sanatorio Americano
EPI_ISL_2964560	2021-03-30	P.6	Institut Pasteur Montevideo
EPI_ISL_2964562	2021-03-30	P.6	Sanatorio Americano
EPI_ISL_2964557	2021-03-31	P.6	Institut Pasteur Montevideo
EPI_ISL_2964566	2021-03-31	P.6	Institut Pasteur Montevideo
EPI_ISL_2964567	2021-03-31	P.6	Sanatorio Americano
EPI_ISL_2964568	2021-04-01	P.6	Sanatorio Americano
EPI_ISL_2427775	2021-04-02	P.6	Médica Uruguaya
EPI_ISL_2964483	2021-04-03	P.6	Sanatorio Americano
EPI_ISL_11799618	2021-04-05	P.6	DLSP, MSP
EPI_ISL_2427554	2021-04-05	P.6	Médica Uruguaya
EPI_ISL_2427575	2021-04-05	P.6	Médica Uruguaya
EPI_ISL_2427591	2021-04-05	P.6	Médica Uruguaya
EPI_ISL_2427529	2021-04-06	P.6	Médica Uruguaya
EPI_ISL_2964702	2021-04-06	P.6	CENUR, Litoral Norte
EPI_ISL_2427553	2021-04-08	P.6	Médica Uruguaya
EPI_ISL_2427710	2021-04-08	P.6	Médica Uruguaya
EPI_ISL_2427718	2021-04-08	P.6	Médica Uruguaya
EPI_ISL_2427726	2021-04-08	P.6	Médica Uruguaya
EPI_ISL_2964570	2021-04-09	P.6	Sanatorio Americano
EPI_ISL_2964589	2021-04-09	P.6	Sanatorio Americano
EPI_ISL_2964590	2021-04-09	P.6	Sanatorio Americano
EPI_ISL_2964593	2021-04-09	P.6	Sanatorio Americano
EPI_ISL_2964597	2021-04-09	P.6	Sanatorio Americano
EPI_ISL_2964625	2021-04-09	P.6	Sanatorio Americano
EPI_ISL_2964626	2021-04-09	P.6	Sanatorio Americano
EPI_ISL_2964634	2021-04-09	P.6	Sanatorio Americano
EPI_ISL_2427716	2021-04-10	P.6	Médica Uruguaya
EPI_ISL_2964569	2021-04-10	P.6	Institut Pasteur Montevideo
EPI_ISL_2964571	2021-04-10	P.6	Sanatorio Americano
EPI_ISL_2964587	2021-04-10	P.6	Sanatorio Americano
EPI_ISL_2964588	2021-04-10	P.6	Institut Pasteur Montevideo
EPI_ISL_2964591	2021-04-10	P.6	Sanatorio Americano
EPI_ISL_2964592	2021-04-10	P.6	Sanatorio Americano
EPI_ISL_2964596	2021-04-10	P.6	Sanatorio Americano
EPI_ISL_2964633	2021-04-10	P.6	Sanatorio Americano
EPI_ISL_2964694	2021-04-10	P.6	CENUR, Litoral Norte
EPI_ISL_2964595	2021-04-12	P.6	Institut Pasteur Montevideo

gisaid_epi_isl	date	ngolin_line	originating_lab
EPI_ISL_2964689	2021-04-12	P.6	Institut Pasteur Montevideo
EPI_ISL_2427722	2021-04-13	P.6	Médica Uruguaya
EPI_ISL_2427752	2021-04-13	P.6	Médica Uruguaya
EPI_ISL_2427774	2021-04-13	P.6	Médica Uruguaya
EPI_ISL_2964687	2021-04-13	P.6	Institut Pasteur Montevideo
EPI_ISL_2964650	2021-04-17	P.6	Institut Pasteur Montevideo
EPI_ISL_2964654	2021-04-17	P.6	Sanatorio Americano
EPI_ISL_2964638	2021-04-18	P.6	Sanatorio Americano
EPI_ISL_2964646	2021-04-18	P.6	Institut Pasteur Montevideo
EPI_ISL_2964651	2021-04-18	P.6	Sanatorio Americano
EPI_ISL_2964672	2021-04-18	P.6	Sanatorio Americano
EPI_ISL_11799566	2021-04-19	P.6	DLSP, MSP
EPI_ISL_2964635	2021-04-19	P.6	Sanatorio Americano
EPI_ISL_2964636	2021-04-19	P.6	Institut Pasteur Montevideo
EPI_ISL_2964637	2021-04-19	P.6	Institut Pasteur Montevideo
EPI_ISL_2964649	2021-04-19	P.6	Institut Pasteur Montevideo
EPI_ISL_2964653	2021-04-19	P.6	Sanatorio Americano
EPI_ISL_2964655	2021-04-19	P.6	Sanatorio Americano
EPI_ISL_2964648	2021-04-20	P.6	Institut Pasteur Montevideo
EPI_ISL_2964690	2021-04-25	P.6	Institut Pasteur Montevideo

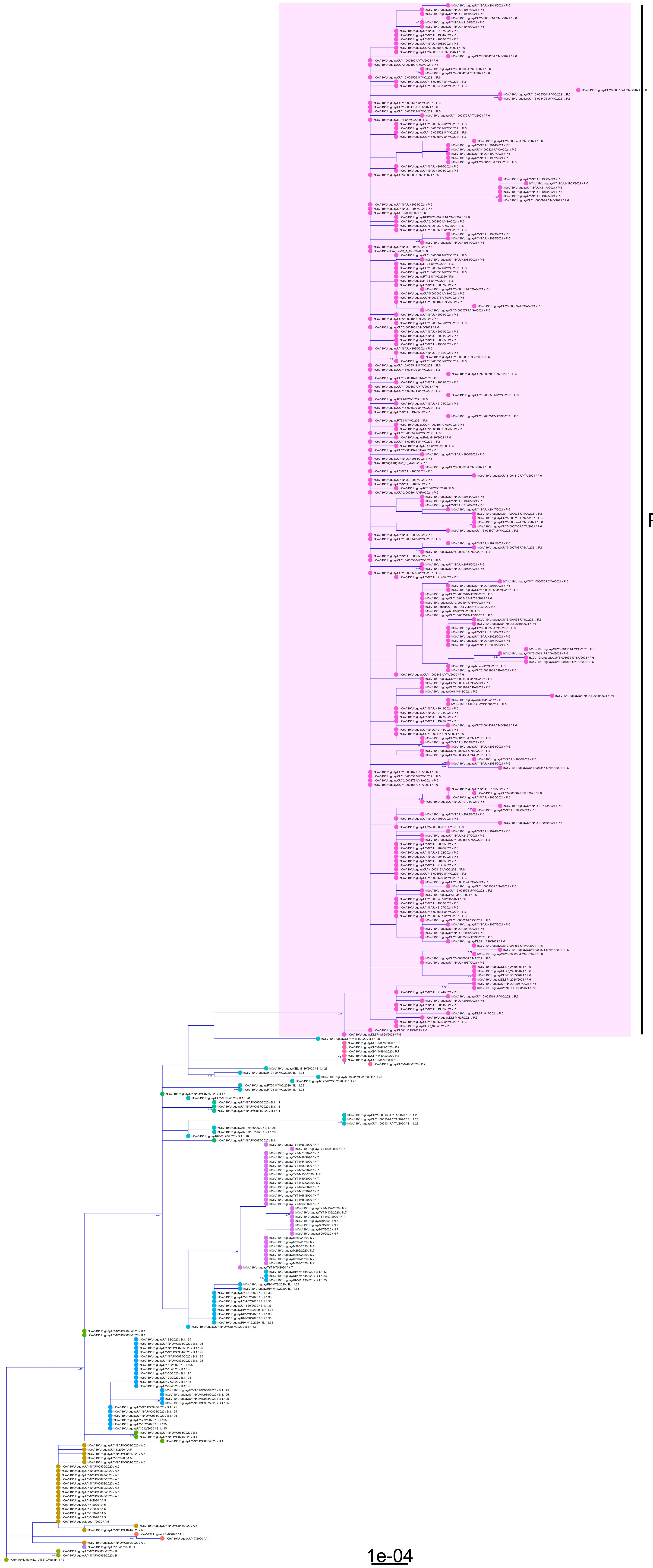

Phylogenetic analysis based on 368 complete severe acute respiratory syndrome coronavirus 2 (SARS-CoV-2) genomes from Uruguay. The maximum-likelihood tree was inferred in Geneious using PhyML (GTR model) with 100-replicates bootstrap to support internal nodes. Pink shadow highlights the P.6 lineage sequences.
